# Identification of Aging-Related Genes Associated with Prognostic Value and Immune Microenvironment Characteristics in Diffuse Large B-Cell Lymphoma

**DOI:** 10.1155/2022/3334522

**Published:** 2022-01-13

**Authors:** Cancan Luo, Han Nie, Li Yu

**Affiliations:** ^1^Department of Hematology, The Second Affiliated Hospital of Nanchang University, Nanchang, China; ^2^Institute of Hematology, Nanchang University, Nanchang, China; ^3^Department of Stem Cell Biology, Atomic Bomb Diseases Institute, Nagasaki University, Japan; ^4^Department of Stem Cell Biology, Nagasaki University Graduate School of Biomedical Sciences, 1-12-4 Sakamoto, Nagasaki 852-8523, Japan

## Abstract

Diffuse large B-cell lymphoma (DLBCL) is a complex invasive tumour that occurs mainly among the elderly. Therefore, we analysed the relationship between ageing-related genes (AG) and DLBCL prognosis. Datasets related to DLBCL and human AGs were downloaded and screened from the Gene Expression Omnibus (GEO) database and HAGR website, respectively. LASSO and Cox regression were used to analyse AGs in the dataset and construct an AG predictive model related to DLBCL prognosis. Gene Ontology and the Kyoto Encyclopedia of Genes and Genomes enrichment were used to analyse the function of the AG predictive model. The immune microenvironment and immune cell infiltration in DLBCL and their relationship with the AG prediction model were also analysed. After the analysis, 118 AGs were identified as genes related to DLBCL prognosis. Using the LASSO and Cox regression analyses, 9 AGs (PLAU, IL7R, MYC, S100B, IGFBP3, NR3C1, PTK2, TBP, and CLOCK) were used to construct an AG prognostic model. In the training and verification sets, this model exhibited excellent predictive ability for the prognosis of patients with DLBCL who have different clinical characteristics. Further analysis revealed that the high- and low-risk groups of the AG prognostic model were significantly correlated with immune cell infiltration and tumour microenvironment in DLBCL. Functional enrichment analysis also showed that the genes in the AG model were associated with immune-related functions and pathways. In conclusion, we constructed an AG model with a strong predictive function in DLBCL, with the ability to predict the prognosis of patients with different clinical features. This model provides new ideas and potential therapeutic targets for the study of the pathogenesis of DLBCL.

## 1. Introduction

Diffuse large B-cell lymphoma (DLBCL) is the most common lymphoid neoplasm in adults, accounting for approximately 30% of non-Hodgkin's lymphomas (NHLs) diagnosed annually. DLBCL exhibits a striking heterogeneity at the clinical, genetic, and molecular levels [1]. The standard rituximab, cyclophosphamide, adriamycin, vincristine, and prednisone (R-CHOP) regimens are the first-line immunochemotherapy regimens for patients with DLBCL; however, 30%-40% of patients remain resistant to R-CHOP and are refractory or experience relapse [2, 3]. The prognosis of DLBCL has been developed from International Prognostic Index (IPI) scores, up to the genetic subtype classification [4]. Considering age, disease stage, serum lactate dehydrogenase (LDH) level, Eastern Cooperative Oncology Group (ECOG), performance status (PS), and the number of extranodal sites, patients with newly diagnosed DLBCL can be stratified into three different risk groups. Furthermore, while recurrent genetic aberrations in individual genes have elucidated oncogenic mechanisms in DLBCL, the progress toward a genetic classification of DLBCL tumours is characterised by genomic aberrations in subtype-specific hallmark genes. The potential clinical utility of this genetic classification is evident from the association of the subtypes with outcomes following R-CHOP therapy [5].

Considering IPI and NCCN-IPI scores, age > 60 years is an adverse prognostic factor for DLBCL. Older patients with DLBCL (≥60 years) are associated with a worse outcome compared to younger patients (<60 years) [6]. Within 2 years after the diagnosis of DLBCL, the standard R-CHOP immunochemotherapy drugs can normalise the life expectancy of young patients, as exhibited by event-free survival rates; however, elderly patients will still experience high mortality [7]. Moreover, it should be noted that the main age group for DLBCL is among the elderly. The median age at the time of diagnosis is 66 years; furthermore, 20.1% of patients are diagnosed between 75 and 84 years of age, 25.0% between 65 and 74 years of age, and 21.2% between 55 and 64 years of age. At present, effective treatment among the elderly remains challenging owing to the adverse invasive biological characteristics of DLBCL, the baseline health status of this patient population, the late toxicity of chemotherapy, and the poor therapeutic effect of the current treatment scheme on elderly patients [8, 9]. Therefore, interest in the understanding of the molecular and genetic pathways dysregulated in ageing and DLBCL has tremendously increased, along with the interest in biomarkers as a quick and quantitative measure in all areas of biomedical research.

Ageing is a process wherein the adaptability and organ function of all organisms decline over time, eventually leading to death [10]. Ageing is often caused by changes in the genome and cells, including tissue degradation and disintegration caused by loss of stem cell renewal ability, genomic instability, telomere wear, metabolic changes, changes in cell communication, cell ageing, loss of protein homeostasis, and epigenetic changes [11]. Notably, B cell immune weakness is a sign of systemic weakness, and ageing may lead to a sharp decline in B cell diversity with age, which could have an important impact on the immune health of the elderly [12]. Because the incidence of DLBCL is mainly among the elderly and considering that the treatment effect for this patient population is poor, we speculate that DLBCL incidence is related to body ageing and cellular immunity. Therefore, because multiple molecular pathways are involved in the ageing process and can contribute to the various aspects of DLBCL, a panel of valid ageing genes (AGs) in DLBCL and its effect on immune function may allow both diagnosis and follow-up in preclinical and clinical settings.

## 2. Materials and Methods

### 2.1. Data Access, Acquisition of AGs, and Setting of Samples

We searched and screened the Gene Expression Omnibus (GEO) database (https://www.ncbi.nlm.nih.gov/geo/) and obtained the gene expression matrix and clinical data of three datasets: GSE10846 (which included tumour specimens and the clinical data of 414 patients with DLBCL from 10 institutions in North America and Europe) [13], GSE11318 (which included tumour specimens and the clinical data of 203 patients with DLBCL from the US National Institutes of Health and the University of Nebraska Medical Center, among others) [14], and GSE32918 (which included tumour specimens and the clinical data of 172 patients with DLBCL from the Yorkshire and Humberside Haematology Network) [15]. We used GSE10846 (included tumour specimens and the clinical data of 414 patients with DLBCL) in the screening of feature genes and as the training set during model construction, whereas GSE11318 and GSE32918 were used as the verification set (details on the original and clinical data of these datasets can be obtained in the cited references). A total of 307 AGs were obtained from HAGR (http://genomics.senescence.info/genes/) [16] (Supplementary Table 1). The expression matrix of the AGs in the training set was extracted and sorted out by using the limma package in R (version 4.0.2).

### 2.2. Screening and Modelling of Prognosis-Related Ageing Genes

Combined with the overall survival (OS) from the clinical information of the training set, prognosis-related AGs were screened using univariate Cox regression analysis. The “glmnet” package in R (version 4.0.2) was used to analyse the prognosis-related AGs through the least absolute shrinkage and selection operator (LASSO) Cox regression analysis, and a predictive model was constructed. The risk score of the model was determined according to the standardised expression level of each gene and the corresponding regression coefficient. The formula for calculating the risk score was as follows:

(1) $$\begin{array}{c} \text{Risk score}=\sum i=1n\text{Coef}\left(i\right)\times x\left(i\right). \end{array}$$

Patients were divided into high- and low-risk groups according to the risk values calculated by the model.

### 2.3. Verification of the Ageing Gene Prognostic Model

The risk score of the training set samples was scored according to the AG prognostic model. Then, principal component analysis (PCA) was performed according to the risk score. The “survminer” package was used to analyse survival in the training set samples scored by the model and to evaluate the prognosis prediction of the samples. The “timeROC” package was used to analyse the receiver-operating characteristic (ROC) curve of the training set to evaluate the model efficiency. Then, univariate and multivariate Cox analyses of the clinical characteristics of the model were performed according to the clinical characteristics. The AG prognostic model was used to score the risk in the two verification sets, and the survival and ROC curve analyses of the validation set were performed using the “survminer” and “timeROC” packages, respectively, to test the model reliability.

### 2.4. GO and KEGG Enrichment Analysis

The genes in the AG prognostic model were analysed using the Gene Ontology (GO) and Kyoto Encyclopedia of Genes and Genomes (KEGG) through the “clusterProfiler4.0” package [17].

### 2.5. Prediction of Clinical Features and Nomogram Construction Using the Ageing Gene Prognostic Model

The AG prognostic model was used to score and group the patients with DLBCL who have different clinical characteristics (age, treatment, ECOG grade, subtype, gender, and stage) and to test the reliability of the grouping. A nomogram was constructed to predict the prognosis of these patients according to the various clinical characteristics.

### 2.6. Relationship between the Ageing Gene Prognostic Model and the Tumour Microenvironment

ESTIMATE [18] was used to score the tumour microenvironment (including tumour purity, immune score, stroma score, and ESTIMATE score) of the training set samples. Differences in the microenvironment between the high- and low-risk groups distinguished through the AG prognostic model were analysed, and survival analysis was performed using the survival time.

### 2.7. Relationship between the Ageing Gene Prognostic Model and the Immune Cell Infiltration and Immune Checkpoint

CIBERSORT [19] was used to score 22 types of immune cell infiltration in the training set samples. The difference in the high- and low-risk groups distinguished through the AG prognostic model was analysed, and survival analysis was performed using the survival time. We also analysed the relationship between the high- and low-risk groups and the 37 immune checkpoints as well as the relationship between immune checkpoints and prognosis.

### 2.8. Statistical Analysis

Survival analysis was performed using the “survminer” package. The ROC curve and ROC analysis were completed using the “timeROC” package. The Cox proportional hazard regression model was used for univariate and multivariate analyses. *P* < 0.05 was considered to have statistical significance, whereas *P* < 0.01 was significant.

## 3. Results

### 3.1. Screening and Modelling of Prognosis-Related Ageing Genes

**Figure 1:**
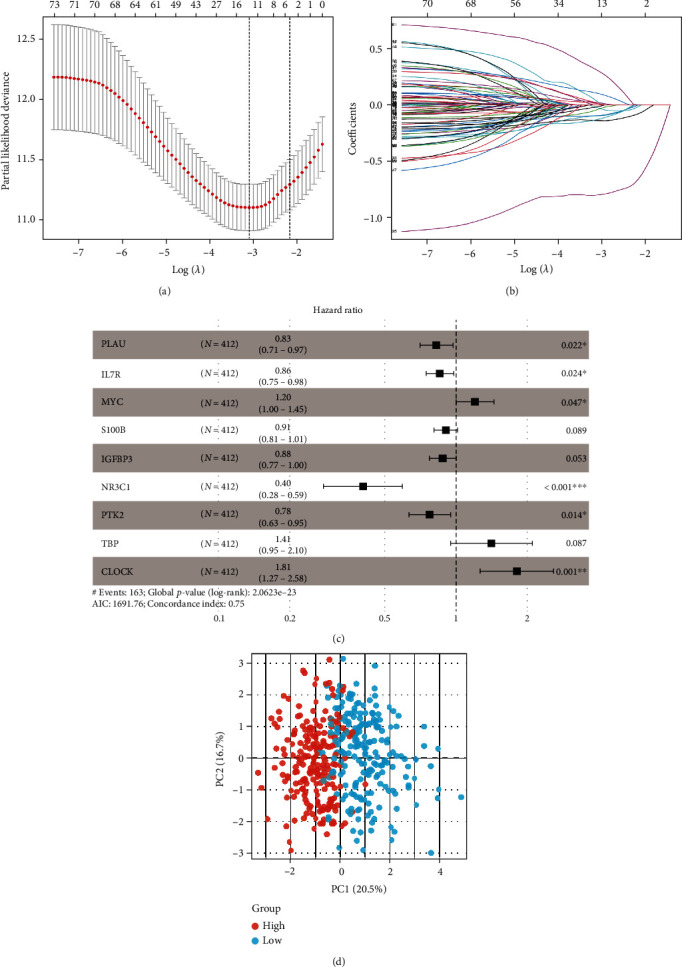
(a) Cross validation for adjusting parameters in the LASSO regression analysis. (b) LASSO regression analysis of the AG prognostic model (each curve represents each prognostic AG included in the construction of the prognostic model). (c) Gene forest map of the AG prediction model. (d) PCA cluster analysis of the training set samples.

Supplementary Figure 1 shows the flow chart of the work. We obtained the expression of 296 AGs using the “limma” package to extract the AGs from the training set GSE10847. Moreover, we obtained 118 AGs related to the prognosis of DLBCL (Supplementary Figure 2) using univariate Cox regression analysis combined with the total survival time in the training set. A model of the AGs related to prognosis was constructed using LASSO regression analysis (Figures 1(a) and 1(b)). A predictive model was also constructed using nine AGs: PLAU, IL7R, MYC, S100B, IGFBP3, NR3C1, PTK2, TBP, and CLOCK (Figure 1(c)). The risk score of the AG prediction model is shown in Table 1. The description of the nine model genes is shown in Table 2.

### 3.2. Verification of the Ageing Gene Prognostic Model

**Figure 2:**
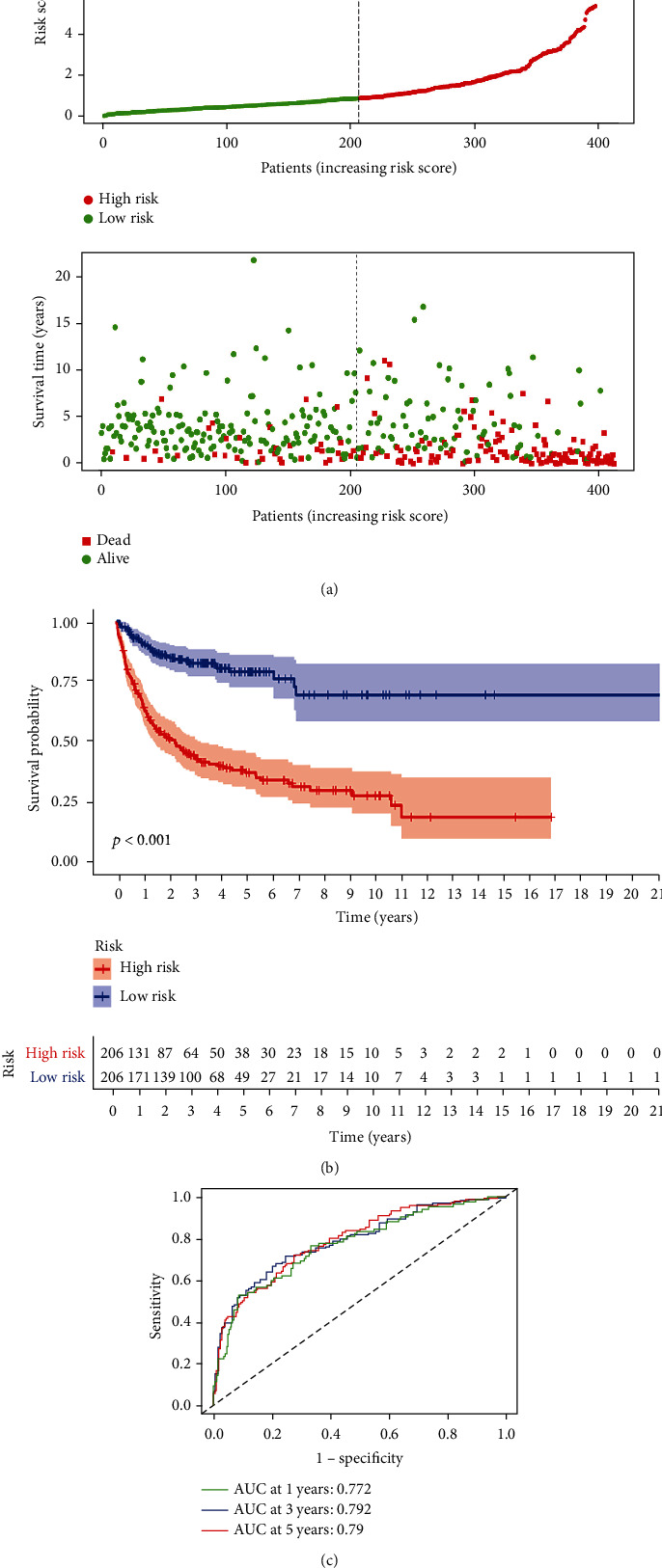
(a) The model divides patients in the training set into low-risk and high-risk groups, and the number of survival and deaths was compared between these two groups. (b) Kaplan-Meier curve between the high-risk and low-risk groups. (c) The subject working curve of the model in the training set.

**Figure 3:**
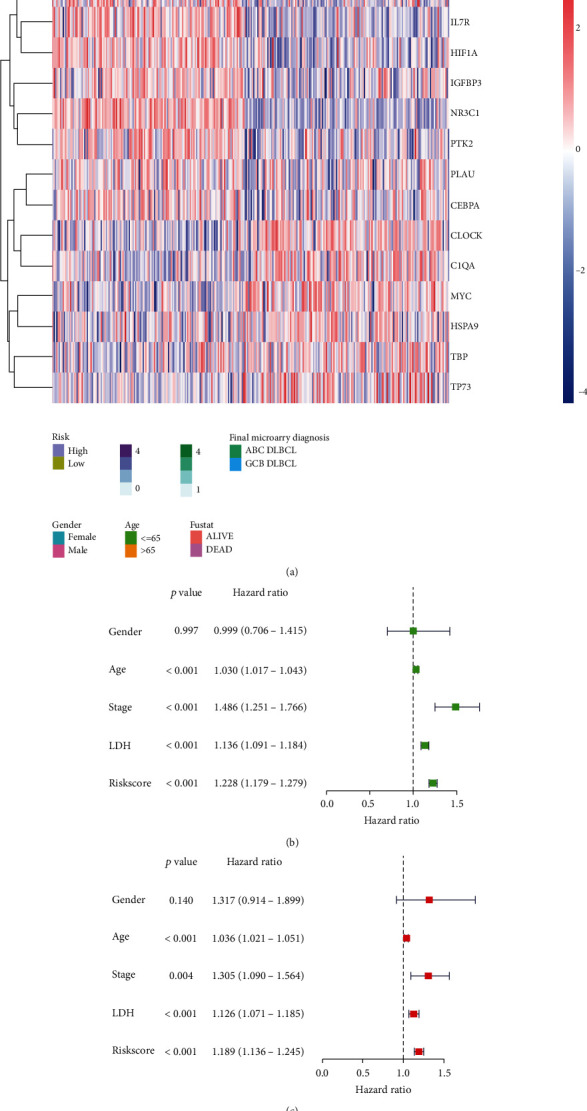
(a) The heatmap of clinical characteristics of the high- and low-risk group in the training focus. (b) Univariate analysis of the clinical characteristics of the training set (which shows whether these clinical features and prognostic models are related to the survival time of patients). (c) Multivariate analysis of the clinical characteristics of the training set (which shows whether these characteristics are still related to the survival time of patients, considering the mutual influence of these clinical features and the prognostic models).

The AG prognostic model was used to score the training set and divide the patients into the high- and low-risk groups. Then, PCA was used to analyse the training set samples, with the results showing that the samples could be well distinguished (Figure 1(d)). The survival and ROC analyses of the high- and low-risk groups in the training set showed that the survival characteristics of the high-risk group were significantly lower than those of the low-risk group (Figures 2(a) and 2(b)). Moreover, the ROC analysis showed that the area under curve (AUC) value of the model for 1, 3, and 5 years was 0.772, 0.792, and 0.79 (Figure 2(c)), respectively, which indicates that the AG prognostic model had high prediction accuracy. The two other DLBCL datasets (GSE11318 and GSE32918) were used as independent validation datasets to verify the performance of the AG prognostic model. Consistent with the results of the training set, the survival characteristics of the high-risk group were significantly lower than those of the low-risk group in both verification sets (Supplementary Figures 3A, 3B, 3D, and 3E). Furthermore, the AUC value of the model in both verification sets was satisfactory (Supplementary Figures 3C and 3F). Univariate and multivariate regression analyses were also performed on the AG prognostic model combined with clinical characteristics. The clinical characteristics of the training set and the heatmaps of the 9 AG expression profiles are shown in Figure 3(a). The univariate and multivariate analyses shown in Figures 3(b) and 3(c) also demonstrate that the model could be used as independent prognostic factors in patients with DLBCL. The clinical features of the verification sets and the heatmaps of the nine AG expression profiles are shown in Supplementary Figures 4A and 4D. Similarly, the univariate and multivariate regression analyses demonstrated that the AG prognostic model could also be used as an independent prognostic factor for patients with DLBCL in both validation sets (Supplementary Figures 4B, 4C, 4E and 4F).

### 3.3. GO and KEGG Enrichment Analyses

**Figure 4:**
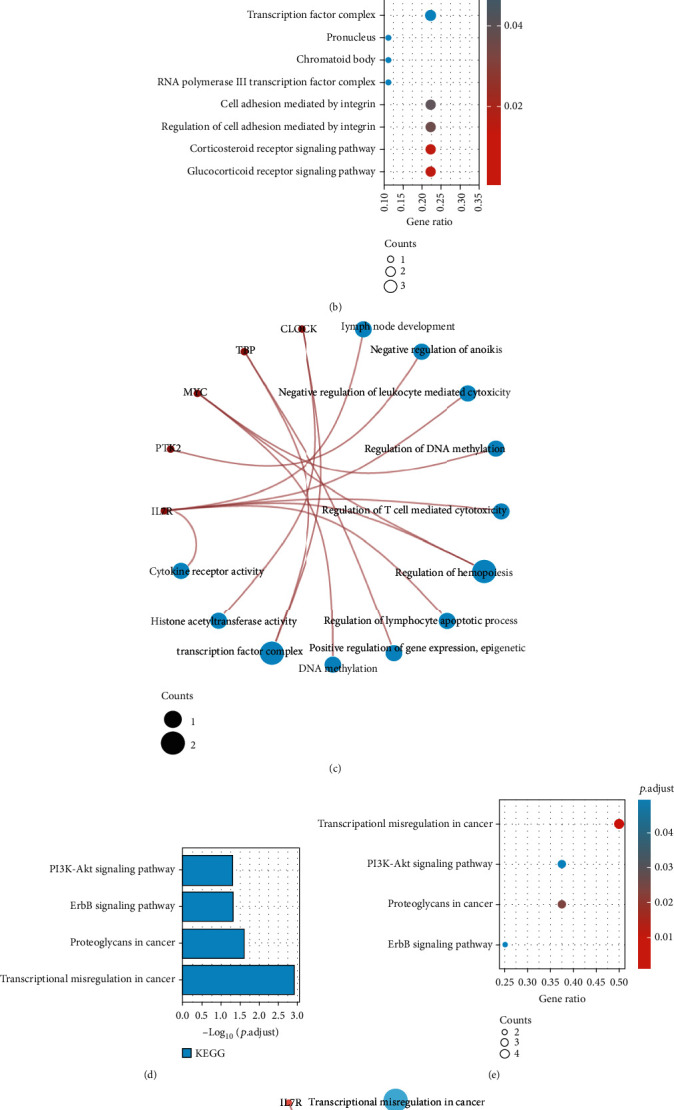
(a) Bubble map of the GO and KEGG enrichment analyses of prognostic model genes. (b) Scatter plot of the GO enrichment analysis of the prognostic model genes. (c) Scatter plot of the KEGG enrichment analysis of the prognostic model genes.

The AG prognostic model genes were analysed through GO and KEGG enrichment analyses. Figures 4(a)–4(c) show that the model gene is related to the development of lymph nodes, the regulation of lymphocyte apoptosis, and the regulation of immune cells. Figures 4(d)–4(f) show that model genes are involved in transcriptional disorders, cell senescence, the PI3K-Akt signal pathway, and the JAK-STAT signal pathway in cancer.

### 3.4. Prediction of Clinical Features and Nomogram Construction Using the Ageing Gene Prognostic Model

**Figure 5:**
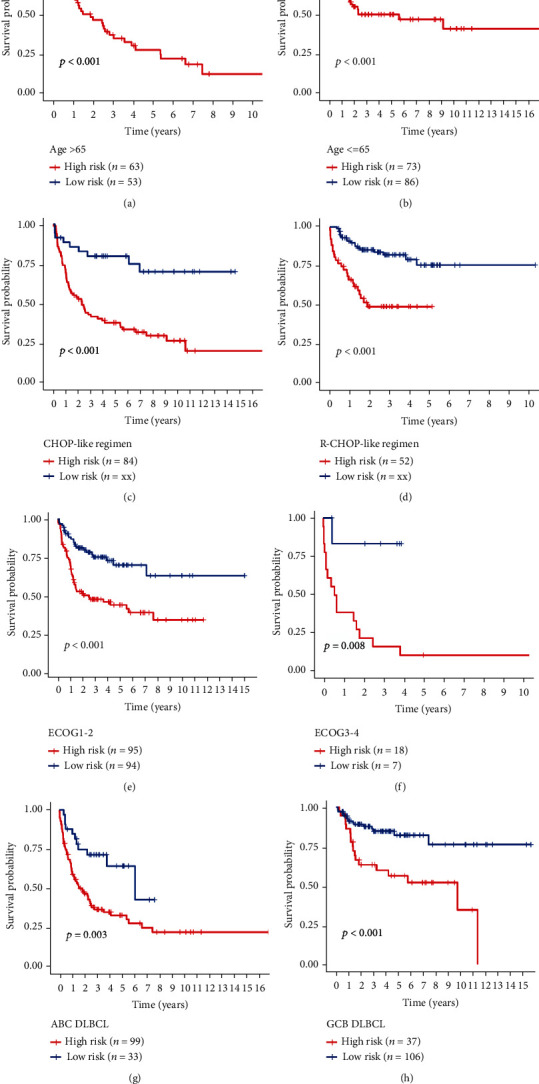
(a–h) Kaplan-Meier curves showing the relationship between age (>65 years, ≤65 years), treatment (CHOP, R-CHOP), ECOG grade (G1-2, G3-4), subtype (ABC, GCB), and survival time of patients with DLBCL in the high- and low-risk groups.

**Figure 6:**
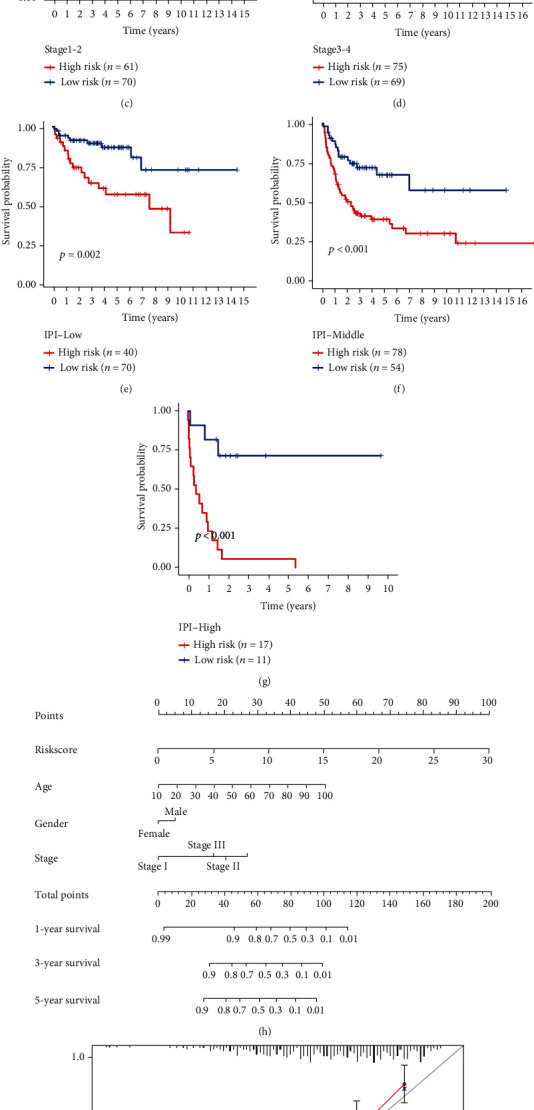
(a–g) Kaplan-Meier curves showing sex (female, male), stage (stage 1-2, stage 3-4), and IPI scores (low, middle, and high) in the high- and low-risk groups. (h) Nomogram constructed according to the prognostic model of patients with DLBCL. (i) The calibration curve used to predict the nomogram.

We tested the stability of the AG prognostic model, and the results show that the model has high accuracy in distinguishing the prognosis of patients with different clinical characteristics, including age (>65 years, ≤65 years), treatment (CHOP, R-CHOP), ECOG grade (0-2 points, 3-4 points), subtype (ABC, GCB), sex (female, male), and grade (G1-2, G3-4) (Figures 5(a)–5(h) and 6(a)–6(d)). Notably, the IPI (International Prognostic Index) score is currently recognised as a prognostic indicator of lymphoma. It scores patients with DLBCL according to age, sites of involvement, clinical stage, ECOG grade, and LDH. The scores are divided into low (0-1), medium (2-3), and high (4-5) risk. However, our model can further distinguish the prognosis of low-, medium-, and high-risk patients (Figures 6(e)–6(g)), suggesting that the AG prognostic model that we constructed has high reliability and accuracy for predicting the prognosis of patients with DLBCL who have different clinical characteristics, and can be combined with the IPI score for more accurate predictions. Combined with the survival analysis, we constructed a nomogram that includes the risk score and other clinicopathological information to predict the survival time of patients in the training set, as shown in Figures 6(h) and 6(i).

### 3.5. Relationship between the Ageing Gene Prognostic Model and the Tumour Microenvironment

**Figure 7:**
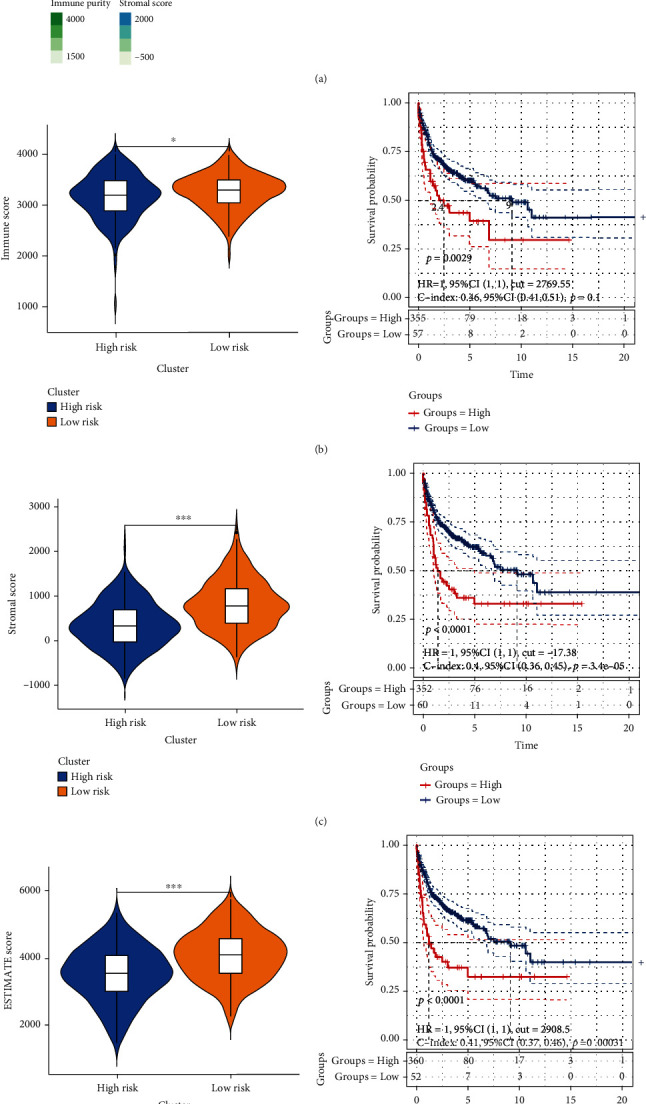
(a) Heatmap of the tumour microenvironment characteristics in the high- and low-risk groups. (b) Immune score map of the high- and low-risk groups and the Kaplan-Meier survival curve of the high- and low-immune score groups (blue represents the high-immune score group, whereas red represents the low-immune score group). (c) The matrix score map of the high- and low-risk groups and the Kaplan-Meier survival curve of the high- and low-matrix groups (blue represents the high matrix score, and red represents the low matrix score). (d) ESTIMATE score chart of the high- and low-risk groups and the Kaplan-Meier survival curve of high and low ESTIMATE scores (blue represents the high ESTIMATE score, and red represents the low ESTIMATE score). (e) Tumour purity score map of the high- and low-risk groups and the Kaplan-Meier survival curve of the high- and low-tumour purity score groups (blue represents the high tumour purity score, whereas red represents the low tumour purity immune score).

Because the ageing of the body is usually accompanied by the decline of immune function and the GO and KEGG enrichment analyses showed that model genes are involved in immune-related functions and pathways, we used the ESTIMATE algorithm to determine stroma and immune scores and to explore the tumour microenvironment differences between the high- and low-risk groups.  Figure 7(a) shows a heatmap of tumour purity, immune score, stroma score, and ESTIMATE score as well as the nine AG expression profiles. Consistent with our initial hypothesis, compared to the low-risk group, the high-risk group had significantly lower immunity, stroma, and ESTIMATE scores. Moreover, the survival analysis also showed that low scores in these three areas predicted a poor prognosis (Figures 7(b)–7(d)). Furthermore, the high-risk group had a high tumour purity score, which also predicted a poor prognosis (Figure 7(e)).

### 3.6. Relationship between the Ageing Gene Prognostic Model and Immune Cell Infiltration

**Figure 8:**
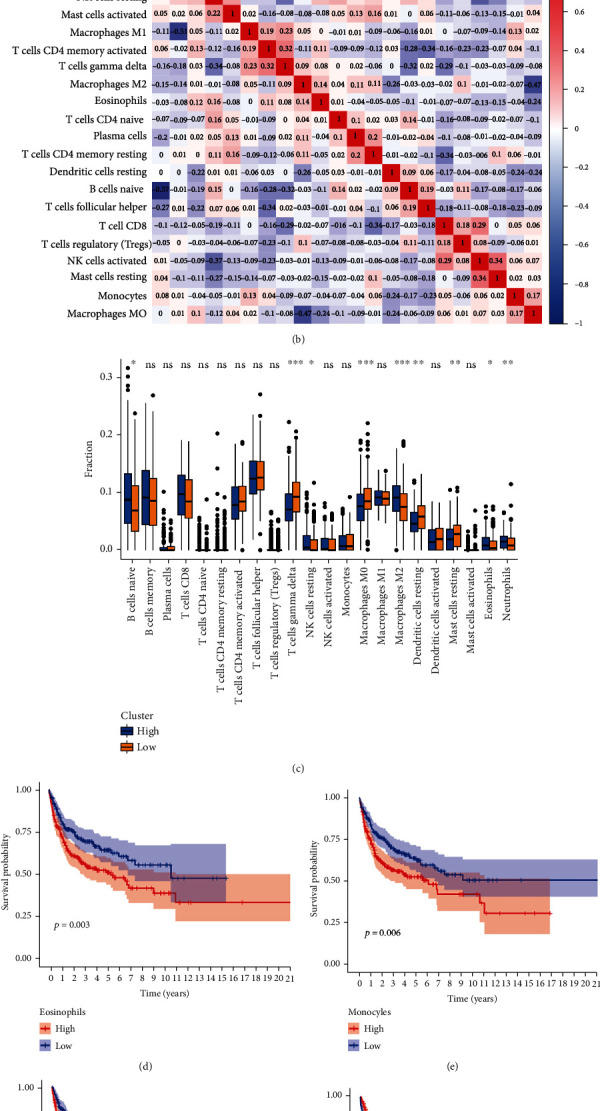
(a) The proportion of cell infiltration in 22 types of immune cells in the high- and low-risk groups. (b) The correlation diagram of the immune cells. (c) The scores of 22 types of immune cells in the high- and low-risk groups (^∗^*P* < 0.05, 0.01, ^∗^*P* < 0.001). (d–i) Kaplan-Meier survival curves of eosinophils, monocytes, M2 macrophages, M0 macrophages, dormant dendritic cells, and delta T cells.

We used the CIBERSORT algorithm to further analyse the difference in immune cell infiltration between the high- and low-risk groups.  Figure 8(a) shows the infiltration of 22 immune cells in the high- and low-risk group. As demonstrated in Figure 8(b), there is a strong negative correlation between macrophage M1 and activated dendritic cells, immature B cells and memory B cells and macrophage M0 and macrophage M2.  Figure 8(c) shows that there is an increased expression of immature B cells, resting NK cells, macrophage M2, eosinophils, and neutrophils in the high-risk group, whereas the expression of delta T cells, macrophages M0, dormant dendritic cells, and resting mast cells increased in the low-risk group. High M2 expression in eosinophils, monocytes, and macrophages was associated with poor prognosis (Figures 8(d)–8(f)), whereas high expression of macrophages M0, dormant dendritic cells, and delta T cells was related with good prognosis (Figures 8(g)–8(i)). This finding suggests that AGs affect the immune function of the body and ultimately affect the prognosis of patients with DLBCL by altering the infiltration of eosinophils, M2 macrophages, delta T cells, M0 macrophages, and dormant dendritic cells.

### 3.7. Relationship between the Ageing Gene Prognostic Model and Immune Checkpoint

**Figure 9:**
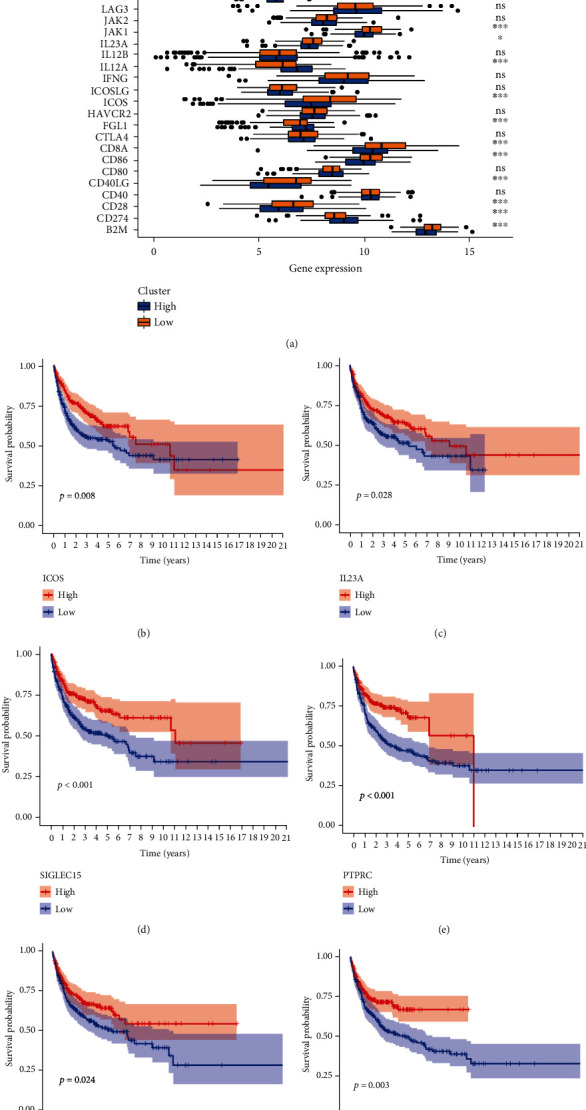
(a) Box chart of the difference distribution of immune checkpoints between the high- and low-risk groups (^∗^*P* < 0.05, ^∗∗^*P* < 0.01, ^∗∗∗^*P* < 0.001). (b–g) Kaplan-Meier survival curves of ICOS, IL23A, SIGLEC15, PTPRC, and LDHA.

**Figure 10:**
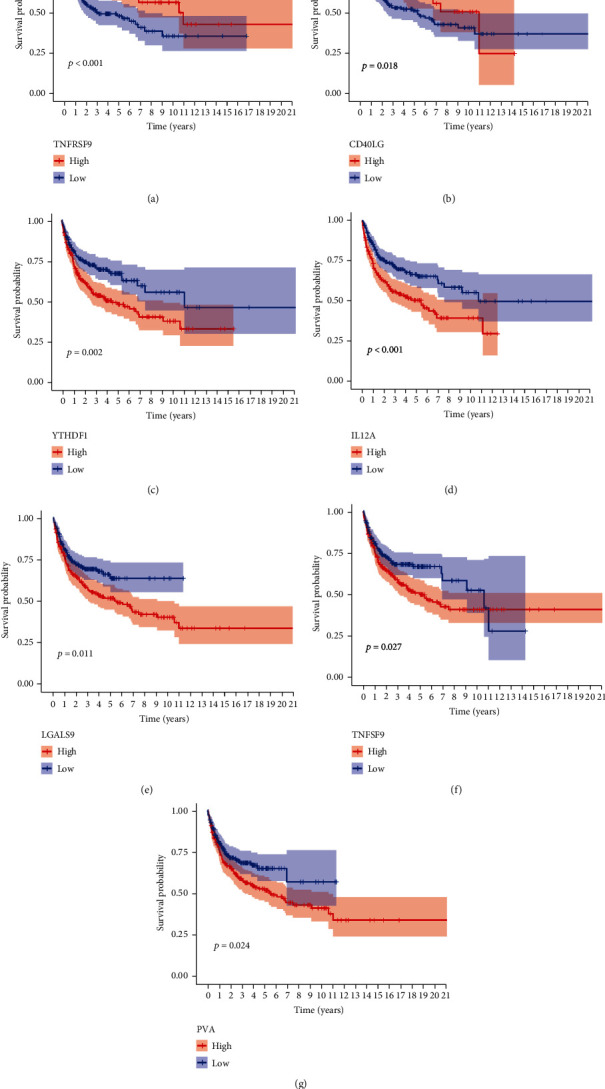
(a–g) Kaplan-Meier survival curves of B2M, TNFRSF9, CD40LG, YTHDF1, IL12A, LGALS9, TNFSF9, and PVR.

Immune checkpoint blockers, which are at the forefront of immunotherapy, are effective in the treatment of many human cancers, especially malignant cancers and chemotherapy-tolerant cancers. Therefore, we analysed the differences in 37 immune checkpoints in DLBCL between the high- and low-risk groups. The results showed that the expression of TNFRSF9, SIGLEC15, PTPRC, PDCD1LG2, LDHA, JAK1, IL23A, ICOS, CD8A, CD86, CD40LG, CD28, and B2M was increased in the low-risk group (Figure 9(a)). The high expression of ICOS, IL23A, SIGLEC15, PTPRC, LDHA, B2M, TNFRSF9, and CD40LG was also associated with a good prognosis (Figures 9(b)–9(g) and 10(a) and 10(b)). Furthermore, the expression of YTHDF1, VTCN1, TNFSF9, TNFSF18, PVR, LGALS9, LDHC, LDHB, LAMA3, IL12A, FGL1, and CD274 was increased in the high-risk group (Figure 9(a)), and the high expression of YTHDF1, IL12A, LGALS9, TNFSF9, and PVR was associated with poor prognosis (Figures 10(c)–10(g)).

## 4. Discussion

The extreme genetic and phenotypic heterogeneity of DLBCL presents a challenge in terms of subtype classification, prognosis prediction, and precision treatment. Ageing is an unavoidable physiological process at present, and the accompanying high-risk disease probability remains an unchangeable fact. In this study, we constructed and verified a new AG prediction model that can well distinguish between the prognosis of patients with high- and low-risk DLBCL. To the best of our knowledge, this study is the first to predict the prognosis of patients with DLBCL characterised by AGs. In addition, we constructed a prognostic nomogram, which can be used to individually estimate the OS probability in patients with DLBCL, help to improve clinical monitoring, and guide the duration of adjuvant chemotherapy and treatment.

In the present study, we determined that the nine central AGs, namely, PLAU, IL7R, MYC, S100B, IGFBP3, NR3C1, PTK2, TBP, and CLOCK, were risk factors related to the prognosis of patients with DLBCL. These AGs were reportedly associated with the underlying mechanisms to facilitate DLBCL or lymphoblastic leukaemia formation and progression. Among them, only MYC is the star protein in DLBCL. Approximately 20%-30% of patients with DLBCL harbour the MYC rearrangement or translocation, which functions as an independent high-risk factor. MYC is also closely related to BCL2, and most patients with refractory DLBCL will exhibit double-hit lymphoma (MYC-BCL2 rearrangement) or double protein expression lymphoma (MYC-BCL2 high expression). The clinical manifestations of this kind of DLBCL are also more aggressive [20–22]. MYC binds to DHX33 and promotes PLAU transcription by directly binding to their promoters, thus promoting cancer cell migration [23]. IL-7R expression in non-GCB-type lymphoma is also significantly higher compared to that in GCB lymphoma [24]. S100B reportedly interacts with p53 involved in many tumours, and S100B (beta) sterically blocks sites of phosphorylation and acetylation on p53 that are important for transcription activation [25]. High S100B expression in antigen-presenting cells is associated with a good prognosis [26]. IGFBP-3 has been proven to play an important role in a variety of tumours. Zhou et al. reported that miR-196b/miR-1290 participates in the antitumour effect of resveratrol by regulating IGFBP3 expression in acute lymphoblastic leukaemia [27]. In in vitro experiments, IGFBP-3 enhancement can induce the apoptosis of breast cancer cells, and the low expression of IGFBP-3 indicates a poor prognosis [28]. In a previous study, high IGFBP-3 expression improved the survival rate of patients with oral squamous cell carcinoma, achieved through NF- *κ*B/IL-6/ROS signalling to promote radiosensitivity [29]. Chan et al. identified the products of NR3C1 as one of the central effectors of the B-lymphoid restriction of glucose and the energy supply, functioning as metabolic gatekeepers by limiting the amount of cellular ATP to levels that are insufficient for malignant transformation [30]. NR3C1 also plays an important role in acute lymphoblastic leukaemia. Studies have confirmed that the deletion of NR3C1 is one of the reasons for the failure of acute lymphoblastic leukaemia induction. In addition, the mutation of NR3C1 may lead to glucocorticoid resistance and lead to treatment failure and recurrence of acute lymphoblastic leukaemia. TBP, a TATA binding protein, is closely related to metabolism. The increased expression of TBP-2 can lead to impaired insulin sensitivity and glucose-induced insulin secretion, as well as *β*-cell apoptosis, leading to diabetes. Furthermore, a transfection experiment showed that TBP-2 expression induces apoptosis in IL-2-independent ATL cells [31]. TBP can directly interact with MYC and regulate the transcriptional process [32–34]. The biological clock has always been thought to be associated with cancer, as in non-Hodgkin's lymphoma. Hoffman et al. have shown that the clock circadian regulator gene can affect the susceptibility to non-Hodgkin's lymphoma by affecting immune regulation, and the overexpression of MYC in U2OS cells (osteosarcoma cells) weakens the clock and in turn promotes cell proliferation [35]. Patients with DLBCL who have high CLOCK expression also show poor overall survival [36].

In our study, the relationship between AG and DLBCL prognosis was initially established, and nine AGs were then used to construct a risk score through the LASSO Cox regression model. This classification breaks DLBCL into two genetic subtypes that exhibit good performance in predicting prognosis both in the training and two validation sets. Compared with patients in the low-risk group, those in the high-risk group typically presented with more aggressive characteristics, advanced stages, and higher mortality (all *P* < 0.05). Furthermore, the ageing risk score exhibited better predictive capability in further distinguishing the low- and high-risk patients as having low, medium, and high risk when the IPI score was considered to predict the prognosis of patients with DLBCL more accurately. It is worth noting that this model was built from large sample datasets and was successfully verified in two small sample datasets, which shows that the performance of this model is very robust and effective.

Results of the GO and KEGG analyses showed that the nine AGs were related to the development of lymph nodes, the process of lymphocyte apoptosis, the regulation of immune cells, and the immune pathway. Ageing is often accompanied by a change in immune function. Therefore, we analysed the relationship between the AG model and immune cell infiltration. The high-risk group had significantly lower immune and matrix scores and a significant increase in the number of dormant NK cells, M2 macrophages, and eosinophils, which represented a low survival rate. NK cells play a key role in preventing haematological malignancies; however, they may enter a state of dysfunction, thus limiting antitumour immunity. Studies have shown that NK cells participate in a large number of metabolic-related transcriptional reprogramming, whereas exposure to fatty acid lymphoma can effectively inhibit NK cell response and cell metabolism [37]. TBP is closely related to the metabolism of the body, and the two can interact with each other and affect DLBCL development. Marchesi et al. have confirmed that the M2 macrophage phenotype is associated with adverse outcomes in patients treated with R-CHOP. Eosinophils are more common in haematological tumours, such as non-Hodgkin's disease and some lymphomas, and may contribute to tumorigenesis and development by promoting angiogenesis and connective tissue formation in adjacent tumours [38–40], which is consistent with our immune cell survival analysis.

The ageing process is inevitably accompanied by changes in the immune system, such as changes in molecular subtypes or expression levels on the surface of immune cells and enhancement or suppression of immune cell function, and these changes may affect the immune monitoring process, including the immune checkpoint [41–43]. At present, immune checkpoint therapy has provided a huge breakthrough for the treatment of lymphomas. Finding reliable predictive biomarkers and potential targets can hence help reduce the side effects of immunosuppressive therapy and expand its applicability to patients with DLBCL. Therefore, we further investigated the relationship between the AG model and the immune checkpoint. The expression of TNFSF9, CD274, and PVR increased in the high-risk group of the AG model. Moreover, the blockers of CD274 (PD-L1) have been widely tested in patients with a variety of lymphomas, and the blockers of PD-L1, nivolumab, and pembrolizumab have been proven effective for the treatment of recurrent or refractory lymphomas [44]. Furthermore, repeated deletions of TNFSF9 have been detected in patients with DLBCL and Burkett's lymphoma [45]. PVR is a member of the laminin-like family, which not only promotes tumour progression and metastasis but also involves immunomodulation. It is also highly upregulated in the tumour cells of many cancer types and is associated with poor prognosis. In addition, the current PVR immune checkpoint inhibitors have been supported by a large number of experiments in preclinical cancer models, including colon cancer, liver cancer, and melanoma, with satisfactory results [46–50]. Our immune checkpoint survival analysis also showed that the high expression of TNFSF9 and PVR was associated with poor prognosis.

Although our study provides new ideas and potential therapeutic targets for the study of the pathogenesis of DLBCL. However, this study has some limitations. This is a retrospective study; therefore, designing a prospective study or obtaining clinical samples and evaluating them with Western blot or immunohistochemistry will be more convincing.

## 5. Conclusion

In conclusion, our study constructed an AG model that can predict the prognosis of patients with DLBCL who have different clinical features. We provide an ageing genetic framework from which to understand its pronounced genetic and clinical heterogeneity as well as the therapeutic responses in subsets of DLBCL tumours defined by shared pathogenesis. This classification breaks DLBCL into two genetic subtypes that differ with respect to gene expression phenotype, oncogenic pathway engagement, tumour microenvironment, and survival rates. In addition, we also explored the relationship between the AG and immune checkpoints in DLBCL and screened out potential immune checkpoints for treatment. This taxonomy provides a roadmap for the prediction and understanding of AGs involving the biological diversity encompassed within the pathological mechanisms of the disease and will likely shed light on the heterogeneous responses of DLBCL to cytotoxic and molecular-targeted therapies.

## Figures and Tables

**Table 1:** Risk score of the AGs prediction model.

ID	Coef	HR	HR.95L	HR.95H	*P* value
PLAU	-0.1877144	0.82885141	0.70623866	0.97275142	0.02155043
IL7R	-0.1559483	0.85560339	0.74739094	0.9794836	0.02379409
MYC	0.18640335	1.20490817	1.00234197	1.44841156	0.04715864
S100B	-0.0981711	0.90649381	0.80965196	1.01491883	0.08855595
IGFBP3	-0.127716	0.88010325	0.77334383	1.00160072	0.05290174
NR3C1	-0.9042901	0.40482918	0.27603495	0.59371708	3.69E-06
PTK2	-0.2531792	0.77632871	0.63465837	0.94962314	0.0137867
TBP	0.34647524	1.41407449	0.95060454	2.10351051	0.08727396
CLOCK	0.59230219	1.80814633	1.2671264	2.5801634	0.00109444

**Table 2:** Genes in prediction model.

Symbol	Name	Gene ID	Uniprot
PLAU	Plasminogen activator, urokinase	5328	UROK_HUMAN
IL7R	Interleukin 7 receptor	3575	IL7RA_HUMAN
MYC	v-myc avian myelocytomatosis viral oncogene homolog	4609	MYC_HUMAN
S100B	S100 calcium binding protein B	6285	S100B_HUMAN
IGFBP3	Insulin-like growth factor binding protein 3	3486	IBP3_HUMAN
NR3C1	Nuclear receptor subfamily 3, group C, member 1 (glucocorticoid receptor)	2908	GCR_HUMAN
PTK2B	Protein tyrosine kinase 2 beta	2185	FAK2_HUMAN
TAF1	TAF1 RNA polymerase II, TATA box-binding protein- (TBP-) associated factor, 250 kDa	6872	TAF1_HUMAN
CLOCK	Clock circadian regulator	9575	CLOCK_HUMAN

## Data Availability

The data used to support the findings of this study are available from the corresponding author upon request.

## Abbreviations

AG: Aging-related genes
GO: Gene Ontology enrichment
KEGG: Kyoto Encyclopedia of Gene and Genome
DLBCL: Diffuse large B cell lymphoma
NHLs: Non-Hodgkin's lymphomas
R-CHOP: Rituximab, cyclophosphamide, adriamycin, vincristine, prednisone
LDH: Lactate dehydrogenase
ECOG: Eastern Cooperative Oncology Group
IPI: International Prognostic Index
OS: Survival time
PCA: Principal component analysis
ROC: Receiver operating characteristic
AUC: Area under curve
TBP: TATA-binding protein.

## Supplementary Materials

Supplementary 1 Supplementary Figure 1: the flow chart for analyzing AG prognostic model in DLBCL.

Supplementary 2 Supplementary Figure 2: univariate Cox analysis of AGs related to the survival time of patients with DLBCL.

Supplementary 3 Supplementary Figure 3: (a) the model divides the patients from the validation set (GSE11318) into the high- and low-risk groups, and the number of survivals and deaths were compared between these groups. (b) The Kaplan-Meier curve for the validation set (GSE11318) divided into the high- and low-risk groups. (c) The receiver operating curve of the model in the validation set (GSE11318). (d) The model divides the patients in the validation set (GSE32918) into the high and low-risk groups, and the number of survivals and deaths were compared between these groups. (e) The Kaplan-Meier curve of the validation set (GSE32918) divided between the high- and low-risk groups. (f) The receiver-operating curve of the model in the validation set (GSE32918).

Supplementary 4 Supplementary Figure 4: (a) heatmap of the clinical characteristics of the high- and low-risk groups in the validation set (GSE11318). (b) Univariate analysis of the clinical features in the validation set (GSE11318). (c) Multivariate analysis of the clinical features in the validation set (GSE11318). (d) Heatmap of clinical characteristics of the high- and low-risk groups in the validation set (GSE32918). (e) Univariate analysis of the clinical features in the validation set (GSE32918). (f) Multivariate analysis of the clinical features in the validation set (GSE32918).

Supplementary 5 Supplementary Table 1: three hundred nine genes related to aging.
