# Supplementary figures and images for: Identification of Aging-Related Genes Associated with Prognostic Value and Immune Microenvironment Characteristics in Diffuse Large B-Cell Lymphoma
Source: Oxid Med Cell Longev. 2022 Jan 13;2022:3334522. doi: 10.1155/2022/3334522 (PMC8777392; doi:10.1155/2022/3334522)

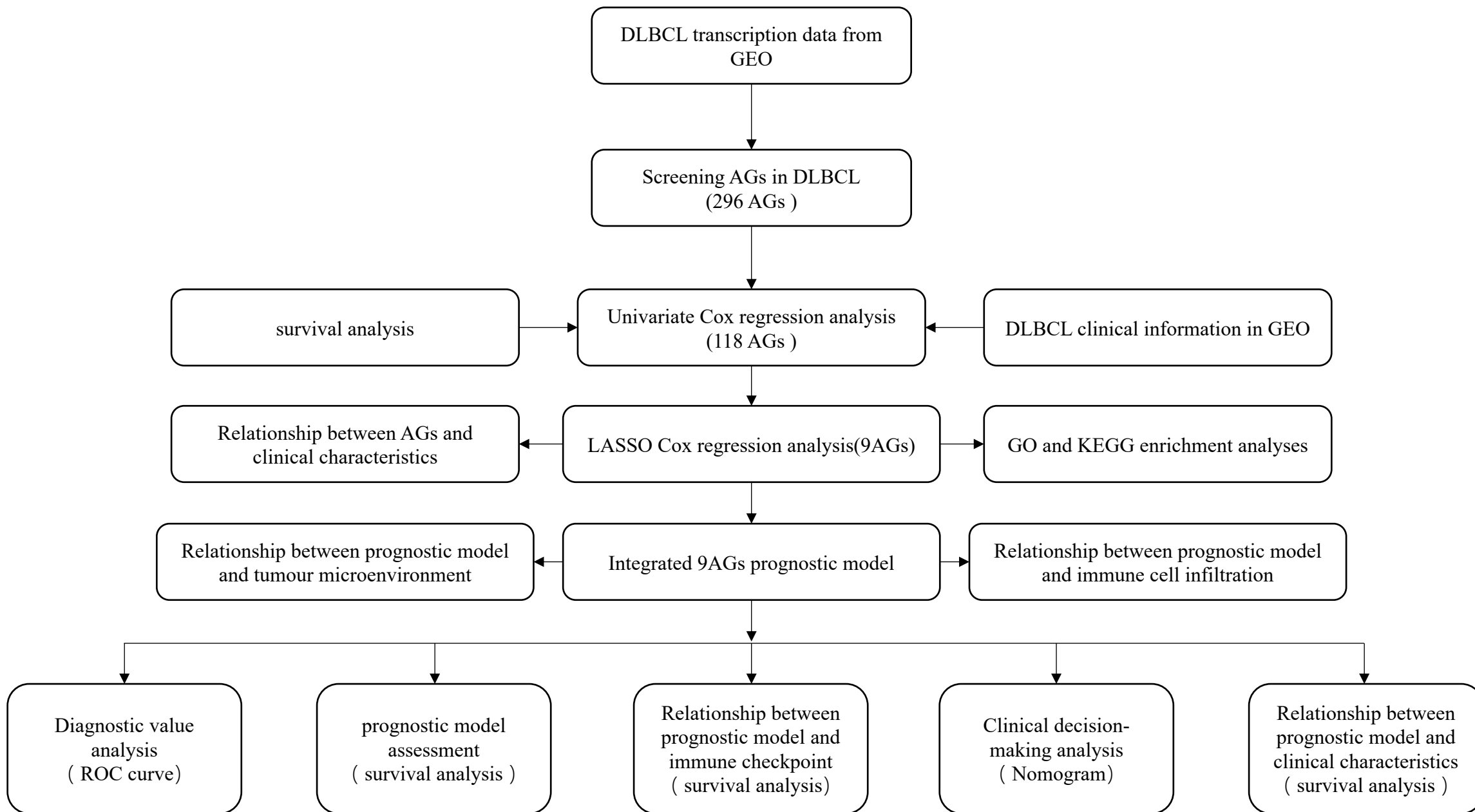

Supplement: Supplementary 1 — Supplementary Figure 1: the flow chart for analyzing AG prognostic model in DLBCL. [file 3334522.f1.pdf]

A

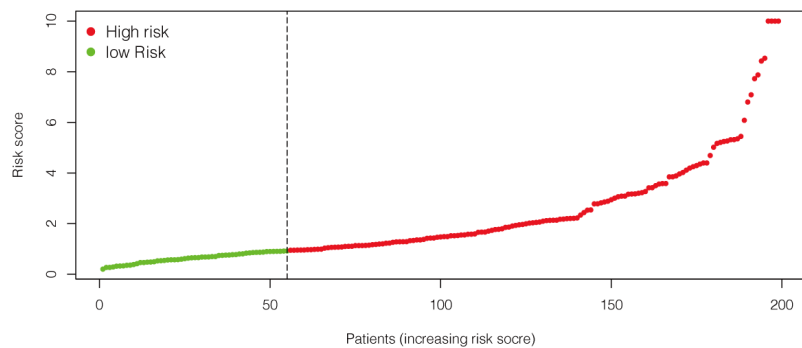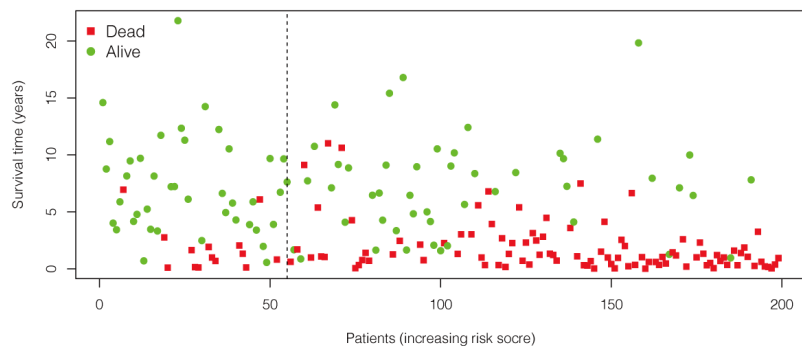

D

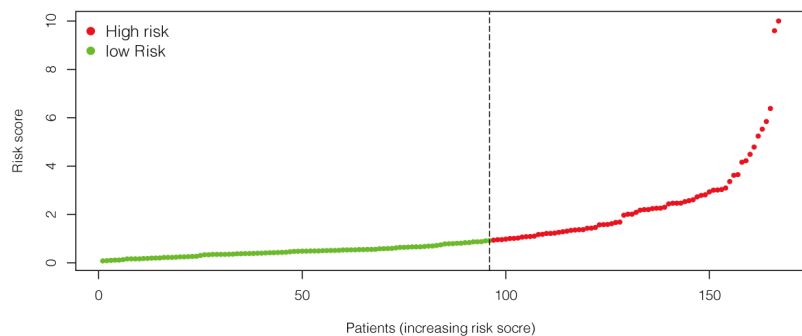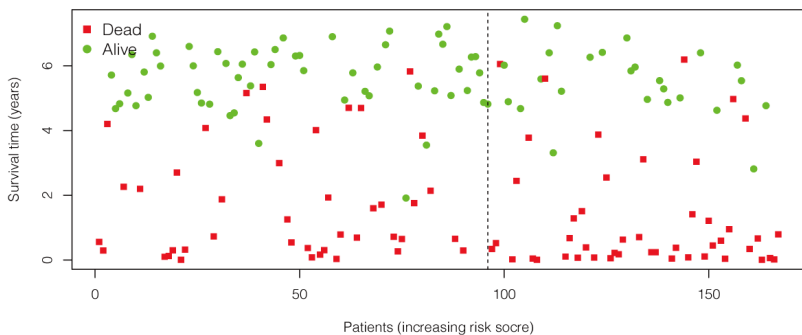

B

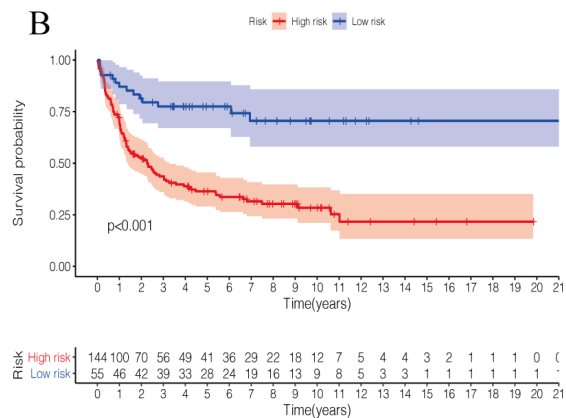

C

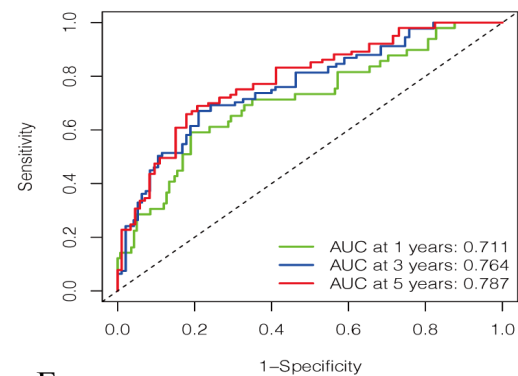

E

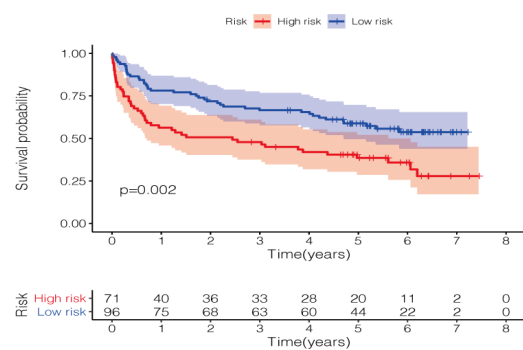

F

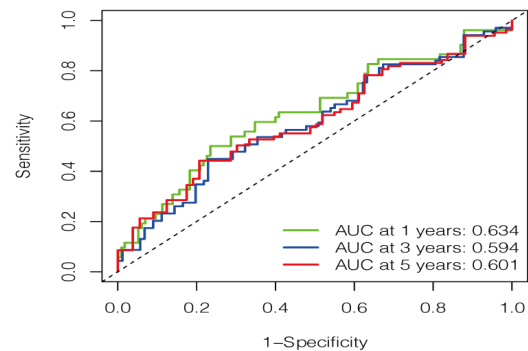

Supplement: Supplementary 3 — Supplementary Figure 3: (a) the model divides the patients from the validation set (GSE11318) into the high- and low-risk groups, and the number of survivals and deaths were compared between these groups. (b) The Kaplan-Meier curve for the validation set (GSE11318) divided into the high- and low-risk groups. (c) The receiver operating curve of the model in the validation set (GSE11318). (d) The model divides the patients in the validation set (GSE32918) into the high and low-risk groups, and the number of survivals and deaths were compared between these groups. (e) The Kaplan-Meier curve of the validation set (GSE32918) divided between the high- and low-risk groups. (f) The receiver-operating curve of the model in the validation set (GSE32918). [file 3334522.f3.pdf]

A

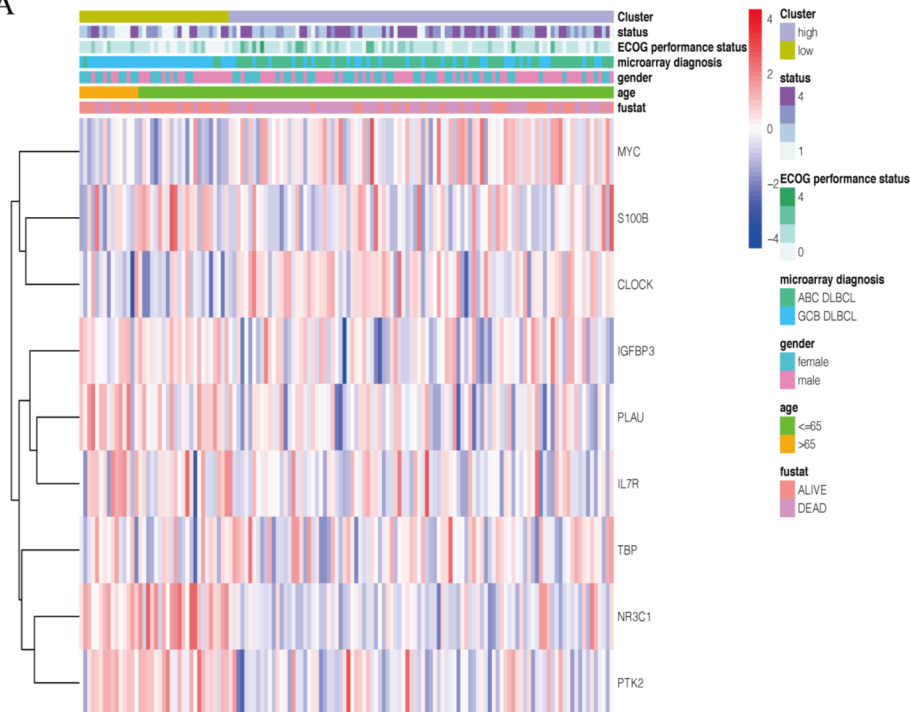

B

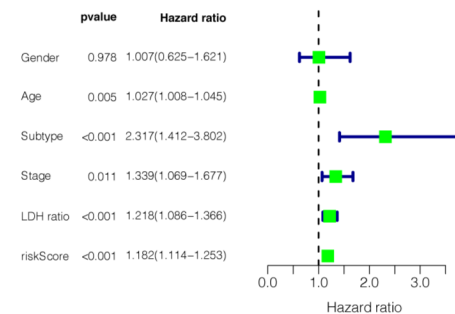

C

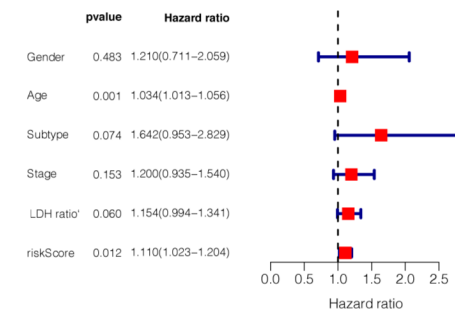

D

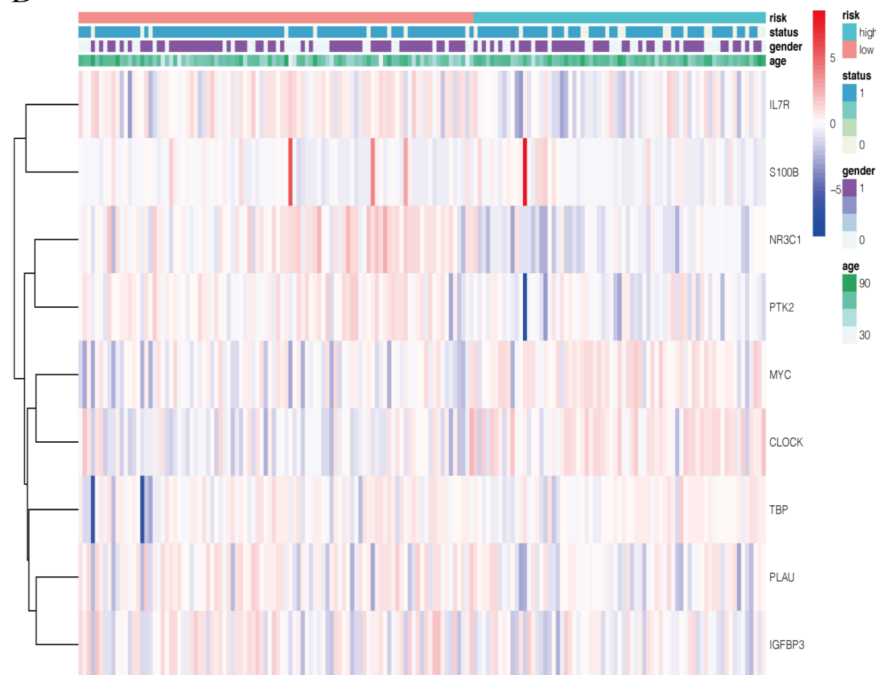

E

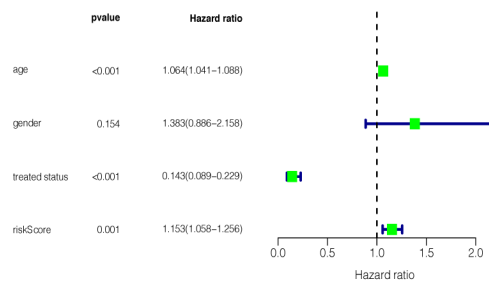

F

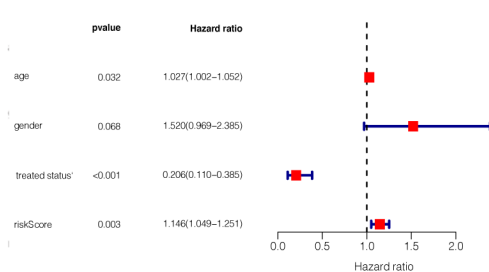

Supplement: Supplementary 4 — Supplementary Figure 4: (a) heatmap of the clinical characteristics of the high- and low-risk groups in the validation set (GSE11318). (b) Univariate analysis of the clinical features in the validation set (GSE11318). (c) Multivariate analysis of the clinical features in the validation set (GSE11318). (d) Heatmap of clinical characteristics of the high- and low-risk groups in the validation set (GSE32918). (e) Univariate analysis of the clinical features in the validation set (GSE32918). (f) Multivariate analysis of the clinical features in the validation set (GSE32918). [file 3334522.f4.pdf]
