# Supplementary material for: Identification of Aging-Related Genes Associated with Prognostic Value and Immune Microenvironment Characteristics in Diffuse Large B-Cell Lymphoma
Source: Oxid Med Cell Longev. 2022 Jan 13;2022:3334522. doi: 10.1155/2022/3334522 (PMC8777392; doi:10.1155/2022/3334522)

	pvalue	Hazard ratio
GHRH	0.003	1.168(1.052−1.296)
TP53	<0.001	1.272(1.105−1.464)
TERT	<0.001	1.270(1.110−1.454)
PLAU	0.007	0.831(0.728−0.950)
ERCC2	<0.001	1.402(1.148−1.711)
E2F1	<0.001	1.400(1.174−1.668)
STAT3	0.039	1.306(1.014−1.684)
STAT5A	0.044	1.218(1.005−1.475)
HDAC3	0.001	1.705(1.228−2.367)
IL7R	<0.001	0.724(0.647−0.811)
IGF1	0.012	0.815(0.694−0.957)
IRS1	0.004	0.810(0.703−0.934)
AKT1	0.011	1.228(1.049−1.438)
PIK3CB	0.023	0.783(0.634−0.967)
HRAS	0.002	1.452(1.147−1.838)
MYC	<0.001	1.520(1.280−1.805)
NBN	0.002	0.623(0.462−0.840)
JUND	<0.001	1.273(1.113−1.457)
PPARA	0.028	1.496(1.045−2.142)
RET	0.006	1.285(1.076−1.535)
TCF3	0.003	1.497(1.152−1.945)
BRCA1	0.002	1.390(1.128−1.713)
PTEN	0.005	0.629(0.455−0.870)
HIF1A	<0.001	0.405(0.300−0.546)
UBB	0.019	0.698(0.516−0.944)
BCL2	0.005	1.291(1.081−1.542)
S100B	<0.001	0.838(0.756−0.928)
VCP	0.001	1.744(1.248−2.436)
POLG	<0.001	1.522(1.186−1.952)
IGFBP3	<0.001	0.749(0.665−0.845)
HSP90AA1	0.005	0.730(0.587−0.909)
NR3C1	<0.001	0.254(0.181−0.356)
EGR1	0.009	0.803(0.681−0.946)
ABL1	<0.001	1.468(1.199−1.798)
TOP1	<0.001	0.814(0.728−0.910)
RAD51	0.026	1.238(1.026−1.493)
UBE2I	0.005	1.424(1.114−1.820)
CEBPA	<0.001	0.770(0.664−0.892)
TGFB1	0.004	1.353(1.102−1.661)
STK11	0.012	1.282(1.056−1.557)
HTT	0.030	1.288(1.025−1.618)
PRKCA	0.013	0.752(0.600−0.941)
ERCC3	0.017	1.397(1.062−1.839)
TERF1	0.006	0.690(0.532−0.897)
ERCC5	0.014	0.640(0.448−0.914)
FAS	0.037	0.844(0.720−0.990)
XRCC6	0.017	1.394(1.061−1.833)
POLD1	0.002	1.412(1.137−1.753)
BAX	0.019	1.285(1.043−1.583)
EMD	0.008	1.335(1.077−1.655)
FOXO1	0.041	0.791(0.632−0.990)
HSF1	0.006	1.270(1.073−1.504)
RECQL4	<0.001	1.318(1.132−1.536)
SOD1	0.030	0.716(0.530−0.968)
FOXM1	0.017	1.216(1.036−1.427)
LRP2	0.041	0.849(0.725−0.994)
AIFM1	0.004	1.353(1.099−1.667)
RELA	0.015	1.304(1.054−1.613)
TOP3B	0.031	1.134(1.012−1.271)
HDAC1	<0.001	0.652(0.508−0.837)
HSPA9	0.004	1.669(1.175−2.370)
GSS	<0.001	1.877(1.383−2.547)
GSTA4	0.017	0.843(0.733−0.970)
GSTP1	0.015	0.789(0.653−0.955)
PCMT1	0.006	0.621(0.443−0.870)
YWHAZ	0.024	0.576(0.357−0.929)
PTK2B	0.036	1.115(1.007−1.234)
PTK2	<0.001	0.652(0.540−0.787)
IL7	0.004	0.859(0.775−0.952)
HMGB1	0.004	0.597(0.421−0.846)
SDHC	0.007	1.711(1.155−2.533)
HESX1	0.042	1.122(1.004−1.253)
PIK3R1	<0.001	0.666(0.562−0.788)
AGPAT2	0.026	1.304(1.032−1.648)
CREB1	<0.001	0.553(0.422−0.726)
TBP	0.009	1.695(1.141−2.519)
HBP1	0.026	0.786(0.636−0.971)
CTGF	<0.001	0.836(0.771−0.908)
SUMO1	0.004	0.656(0.492−0.875)
MTOR	0.015	1.246(1.043−1.487)
DLL3	0.027	1.258(1.027−1.540)
DBN1	0.002	1.304(1.104−1.541)
ATR	0.002	1.524(1.166−1.993)
UCP2	0.001	1.329(1.117−1.580)
GCLC	<0.001	1.530(1.234−1.898)
GCLM	0.004	0.760(0.632−0.914)
SIRT6	<0.001	1.563(1.214−2.012)
CSNK1E	0.001	1.267(1.100−1.460)
STUB1	0.046	1.506(1.006−2.255)
CHEK2	0.025	1.279(1.032−1.585)
ARHGAP1	0.007	1.365(1.087−1.713)
CDC42	0.001	0.625(0.469−0.835)
ARNTL	<0.001	0.757(0.647−0.886)
CLOCK	<0.001	2.348(1.658−3.325)
PPARGC1A	0.031	1.172(1.014−1.354)
GPX4	0.043	0.741(0.555−0.990)
EFEMP1	0.021	0.822(0.697−0.971)
ERCC4	0.003	1.346(1.106−1.638)
SIRT7	0.006	1.436(1.108−1.862)
SIRT3	0.011	1.344(1.069−1.689)
NCOR2	0.019	1.319(1.047−1.663)
BAK1	<0.001	1.862(1.434−2.418)
IGFBP2	<0.001	0.863(0.797−0.934)
PYCR1	0.004	1.293(1.084−1.541)
TP73	<0.001	1.278(1.125−1.453)
NFE2L2	<0.001	0.699(0.579−0.844)
PDGFRA	0.016	0.805(0.675−0.960)
C1QA	<0.001	1.219(1.123−1.324)
CDKN2B	<0.001	0.698(0.592−0.823)
EIF5A2	0.002	0.807(0.704−0.924)
MT1E	0.046	1.122(1.002−1.257)
GSK3A	<0.001	1.419(1.186−1.697)
GRN	0.028	1.234(1.023−1.489)
SPRTN	0.002	0.708(0.568−0.883)
CTF1	<0.001	1.244(1.100−1.408)
NFE2L1	0.014	1.419(1.074−1.874)
IFNB1	0.016	1.180(1.031−1.350)
GDF11	0.005	1.391(1.107−1.749)

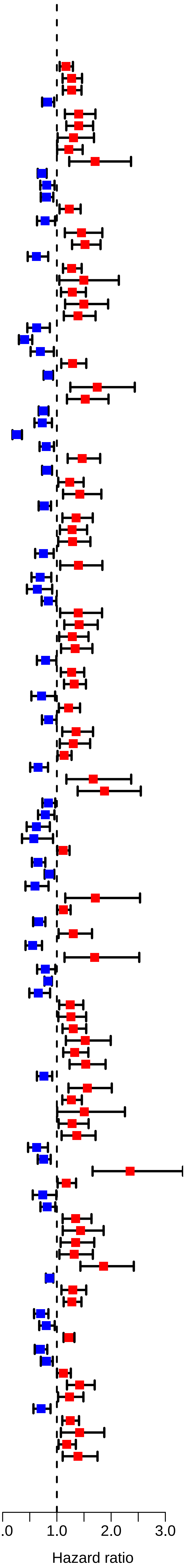

Supplement: Supplementary 2 — Supplementary Figure 2: univariate Cox analysis of AGs related to the survival time of patients with DLBCL. [file 3334522.f2.pdf]
