# Supplementary material for: Identification of Aging-Related Genes Associated with Prognostic Value and Immune Microenvironment Characteristics in Diffuse Large B-Cell Lymphoma
Source: Oxid Med Cell Longev. 2022 Jan 13;2022:3334522. doi: 10.1155/2022/3334522 (PMC8777392; doi:10.1155/2022/3334522)
Supplement: Supplementary 5 — Supplementary Table 1: three hundred nine genes related to aging. [file 3334522.f5.pdf]

Supplementary Table . Three hundred seven genes related to aging.				
**ID**	**Symbol**	**Name**	**Gene id**	**Uniprot**
1	GHR	growth hormone receptor	2690	GHR_HUMAN
2	GHRH	growth hormone releasing hormone	2691	SLIB_HUMAN
3	SHC1	SHC (Src homology 2 domain	6464	SHC1_HUMA
4	POU1F1	POU class 1 homeobox 1	5449	PIT1_HUMAN
5	PROP1	PROP paired-like homeobox 1	5626	PROP1_HUMA
6	TP53	tumor protein p53	7157	P53_HUMAN
7	TERC	telomerase RNA component	7012	TERC
8	TERT	telomerase reverse transcriptase	7015	TERT_HUMA
9	ATM	ATM serine/threonine kinase	472	ATM_HUMAN
10	PLAU	plasminogen activator, urokinase	5328	UROK_HUMA
11	ERCC2	excision repair cross-complementation	2068	ERCC2_HUMA
12	ERCC8	excision repair cross-complementation	1161	ERCC8_HUMA
13	WRN	Werner syndrome, RecQ helicase-like	7486	WRN_HUMAN
14	LMNA	lamin A/C	4000	LMNA_HUMA
15	IGF1R	insulin-like growth factor 1 receptor	3480	IGF1R_HUMA
16	TXN	thioredoxin	7295	THIO_HUMA
17	KL	klotho	9365	KLOT_HUMA
18	E2F1	E2F transcription factor 1	1869	E2F1_HUMAN
19	PTPN11	protein tyrosine phosphatase, non-	5781	PTN11_HUMA
20	NFKB2	nuclear factor of kappa light polypeptide	4791	NFKB2_HUM
21	STAT5B	signal transducer and activator of	6777	STA5B_HUMA
22	STAT3	signal transducer and activator of	6774	STAT3_HUMA
23	STAT5A	signal transducer and activator of	6776	STA5A_HUM
24	NRG1	neuregulin 1	3084	NRG1_HUMA
25	HDAC3	histone deacetylase 3	8841	HDAC3_HUM
26	GH1	growth hormone 1	2688	SOMA_HUMA
27	IL7R	interleukin 7 receptor	3575	IL7RA_HUMA
28	IGF1	insulin-like growth factor 1	3479	IGF1
29	IGF2	insulin-like growth factor 2	3481	IGF2_HUMAN
30	INS	insulin	3630	INS_HUMAN
31	NGF	nerve growth factor (beta polypeptide)	4803	NGF_HUMAN
32	IRS1	insulin receptor substrate 1	3667	IRS1_HUMAN
33	PTPN1	protein tyrosine phosphatase, non-	5770	PTN1_HUMA
34	IRS2	insulin receptor substrate 2	8660	IRS2_HUMAN
35	AKT1	v-akt murine thymoma viral oncogene	207	AKT1_HUMA
36	PIK3CB	phosphatidylinositol-4,5-bisphosphate 3-	5291	PK3CB_HUM
37	NGFR	nerve growth factor receptor	4804	TNR16_HUMA
38	HRAS	Harvey rat sarcoma viral oncogene	3265	RASH_HUMA

39	MYC	v-myc avian myelocytomatosis viral	4609	MYC_HUMAN
40	EGFR	epidermal growth factor receptor	1956	EGFR_HUMA
41	ERBB2	erb-b2 receptor tyrosine kinase 2	2064	ERBB2_HUMA
42	INSR	insulin receptor	3643	INSR_HUMAN
43	NCOR1	nuclear receptor corepressor 1	9611	NCOR1_HUM
44	NBN	nibrin	4683	NBN_HUMAN
45	JUND	jun D proto-oncogene	3727	JUND_HUMA
46	IL2	interleukin 2	3558	IL2_HUMAN
47	PDGFB	platelet-derived growth factor beta	5155	PDGFB_HUM
48	EGF	epidermal growth factor	1950	EGF_HUMAN
49	IL2RG	interleukin 2 receptor, gamma	3561	IL2RG_HUMA
50	FOS	FBJ murine osteosarcoma viral oncogene	2353	FOS_HUMAN
51	PDGFRB	platelet-derived growth factor receptor,	5159	PGFRB_HUM
52	EPOR	Erythropoietin receptor	2057	EPOR_HUMA
53	SST	somatostatin	6750	SMS_HUMAN
54	PRKCD	protein kinase C, delta	5580	KPCD_HUMA
55	PPARA	peroxisome proliferator-activated receptor	5465	PPARA_HUM
56	RET	ret proto-oncogene	5979	RET_HUMAN
57	PLCG2	phospholipase C, gamma 2	5336	PLCG2_HUMA
58	PEX5	peroxisomal biogenesis factor 5	5830	PEX5_HUMA
59	TCF3	transcription factor 3	6929	TFE2_HUMAN
60	PARP1	poly (ADP-ribose) polymerase 1	142	PARP1_HUMA
61	BRCA1	breast cancer 1, early onset	672	BRCA1_HUM
62	PIN1	peptidylprolyl cis/trans isomerase,	5300	PIN1_HUMAN
63	PTEN	phosphatase and tensin homolog	5728	PTEN_HUMA
64	CREBBP	CREB binding protein	1387	CBP_HUMAN
65	HIF1A	hypoxia inducible factor 1, alpha subunit	3091	HIF1A_HUMA
66	UBB	ubiquitin B	7314	UBIQ_HUMA
67	RPA1	replication protein A1, 70kDa	6117	RFA1_HUMA
68	BLM	Bloom syndrome, RecQ helicase-like	641	BLM_HUMAN
69	BCL2	B-cell CLL/lymphoma 2	596	BCL2_HUMA
70	S100B	S100 calcium binding protein B	6285	S100B_HUMA
71	VCP	valosin containing protein	7415	TERA_HUMA
72	POLG	polymerase (DNA directed), gamma	5428	DPOG1_HUM
73	IGFBP3	insulin-like growth factor binding protein	3486	IBP3_HUMAN
74	HSP90AA	heat shock protein 90kDa alpha	3320	HS90A_HUMA
75	NR3C1	nuclear receptor subfamily 3, group C,	2908	GCR_HUMAN
76	EGR1	early growth response 1	1958	EGR1_HUMA
77	VEGFA	vascular endothelial growth factor A	7422	VEGFA_HUM
78	ABL1	ABL proto-oncogene 1, non-receptor	25	ABL1_HUMA

79	BRCA2	breast cancer 2, early onset	675	BRCA2_HUM
80	TOP2A	topoisomerase (DNA) II alpha	7153	TOP2A_HUM
81	TOP2B	topoisomerase (DNA) II beta	7155	TOP2B_HUMA
82	NFKB1	nuclear factor of kappa light polypeptide	4790	NFKB1_HUM
83	TOP1	topoisomerase (DNA) I	7150	TOP1_HUMA
84	RAD51	RAD51 recombinase	5888	RAD51_HUM
85	UBE2I	ubiquitin-conjugating enzyme E2I	7329	UBC9_HUMA
86	TNF	tumor necrosis factor	7124	TNFA_HUMA
87	PDPK1	3-phosphoinositide dependent protein	5170	PDPK1_HUM
88	CEBPA	CCAAT/enhancer binding protein	1050	CEBPA_HUM
89	CEBPB	CCAAT/enhancer binding protein	1051	CEBPB_HUM
90	MXI1	MAX interactor 1, dimerization protein	4601	MXI1_HUMA
91	TGFB1	transforming growth factor, beta 1	7040	TGFB1_HUMA
92	ERCC6	excision repair cross-complementation	2074	ERCC6_HUMA
93	STK11	serine/threonine kinase 11	6794	STK11_HUMA
94	EP300	E1A binding protein p300	2033	EP300_HUMA
95	APTX	aprataxin	54840	APTX_HUMA
96	PML	promyelocytic leukemia	5371	PML_HUMAN
97	GSK3B	glycogen synthase kinase 3 beta	2932	GSK3B_HUM
98	HTT	huntingtin	3064	HD_HUMAN
99	PRKCA	protein kinase C, alpha	5578	KPCA_HUMA
100	SSTR3	somatostatin receptor 3	6753	SSR3_HUMAN
101	HELLS	helicase, lymphoid-specific	3070	HELLS_HUMA
102	APOC3	apolipoprotein C-III	345	APOC3_HUM
103	EEF2	eukaryotic translation elongation factor 2	1938	EF2_HUMAN
104	ERCC3	excision repair cross-complementation	2071	ERCC3_HUMA
105	TERF1	telomeric repeat binding factor (NIMA-	7013	TERF1_HUMA
106	PRKDC	protein kinase, DNA-activated, catalytic	5591	PRKDC_HUM
107	CAT	catalase	847	CATA_HUMA
109	ERCC5	excision repair cross-complementation	2073	ERCC5_HUMA
110	AR	androgen receptor	367	ANDR_HUMA
111	GTF2H2	general transcription factor IIH,	2966	TF2H2_HUMA
112	XRCC5	X-ray repair complementing defective	7520	XRCC5_HUM
113	PCNA	proliferating cell nuclear antigen	5111	PCNA_HUMA
114	FEN1	flap structure-specific endonuclease 1	2237	FEN1_HUMA
115	FAS	Fas cell surface death receptor	355	TNR6_HUMA
116	TERF2	telomeric repeat binding factor 2	7014	TERF2_HUMA
117	XRCC6	X-ray repair complementing defective	2547	KU70_HUMA
118	POLD1	polymerase (DNA directed), delta 1,	5424	DPOD1_HUM
119	BAX	BCL2-associated X protein	581	BAX_HUMAN

120	RB1	retinoblastoma 1	5925	RB_HUMAN
121	EMD	emerin	2010	EMD_HUMAN
122	GRB2	growth factor receptor-bound protein 2	2885	GRB2_HUMA
123	FOXO3	forkhead box O3	2309	FOXO3_HUM
124	FOXO1	forkhead box O1	2308	FOXO1_HUM
125	HSF1	heat shock transcription factor 1	3297	HSF1_HUMA
126	XPA	xeroderma pigmentosum,	7507	XPA_HUMAN
127	MSRA	methionine sulfoxide reductase A	4482	MSRA_HUMA
128	RECQL4	RecQ helicase-like 4	9401	RECQ4_HUM
129	SOD2	superoxide dismutase 2, mitochondrial	6648	SODM_HUMA
130	SOD1	superoxide dismutase 1, soluble	6647	SODC_HUMA
131	FOXM1	forkhead box M1	2305	FOXM1_HUM
132	COQ7	coenzyme Q7 homolog, ubiquinone	10229	COQ7_HUMA
133	CACNA1	calcium channel, voltage-dependent, P/Q	773	CAC1A_HUM
134	LRP2	low density lipoprotein receptor-related	4036	LRP2_HUMAN
135	AIFM1	apoptosis-inducing factor,	9131	AIFM1_HUMA
136	UCHL1	ubiquitin carboxyl-terminal esterase L1	7345	UCHL1_HUM
137	APP	amyloid beta (A4) precursor protein	351	A4_HUMAN
138	APOE	apolipoprotein E	348	APOE_HUMA
139	A2M	alpha-2-macroglobulin	2	A2MG_HUMA
140	SNCG	synuclein, gamma (breast cancer-specific	6623	SYUG_HUMA
141	PRDX1	peroxiredoxin 1	5052	PRDX1_HUM
142	PON1	paraoxonase 1	5444	PON1_HUMA
143	RELA	v-rel avian reticuloendotheliosis viral	5970	TF65_HUMAN
144	IL6	interleukin 6	3569	IL6_HUMAN
145	RGN	regucalcin	9104	RGN_HUMAN
146	ATP5O	ATP synthase, H+ transporting,	539	ATPO_HUMA
147	RAD52	RAD52 homolog, DNA repair protein	5893	RAD52_HUM
148	TOP3B	topoisomerase (DNA) III beta	8940	TOP3B_HUMA
149	ERCC1	excision repair cross-complementation	2067	ERCC1_HUMA
150	SIRT1	sirtuin 1	23411	SIR1_HUMAN
151	HDAC1	histone deacetylase 1	3065	HDAC1_HUM
152	HSPA9	heat shock 70kDa protein 9 (mortalin)	3313	GRP75_HUMA
153	GPX1	glutathione peroxidase 1	2876	GPX1_HUMA
154	GSR	glutathione reductase	2936	GSHR_HUMA
155	GSS	glutathione synthetase	2937	GSHB_HUMA
156	GSTA4	glutathione S-transferase alpha 4	2941	GSTA4_HUM
157	GSTP1	glutathione S-transferase pi 1	2950	GSTP1_HUMA
158	MT-CO1	mitochondrially encoded cytochrome c	4512	COX1_HUMA
159	HSPD1	heat shock 60kDa protein 1 (chaperonin)	3329	CH60_HUMA

160	HSPA1A	heat shock 70kDa protein 1A	3303	HS71A_HUMA
161	HSPA1B	heat shock 70kDa protein 1B	3304	HSPA1B
162	PCMT1	protein-L-isoaspartate (D-aspartate) O-	5110	PIMT_HUMA
163	MAPK8	mitogen-activated protein kinase 8	5599	MK08_HUMA
164	YWHAZ	tyrosine 3-monooxygenase/tryptophan 5-	7534	1433Z_HUMA
165	PTK2B	protein tyrosine kinase 2 beta	2185	FAK2_HUMA
166	PTK2	protein tyrosine kinase 2	5747	FAK1_HUMA
167	IL7	interleukin 7	3574	IL7_HUMAN
168	MAPK14	mitogen-activated protein kinase 14	1432	MK14_HUMA
169	FGFR1	fibroblast growth factor receptor 1	2260	FGFR1_HUMA
170	SP1	Sp1 transcription factor	6667	SP1_HUMAN
171	FLT1	fms-related tyrosine kinase 1	2321	VGFR1_HUM
172	JUN	jun proto-oncogene	3725	JUN_HUMAN
173	MED1	mediator complex subunit 1	5469	MED1_HUMA
174	MAPK9	mitogen-activated protein kinase 9	5601	MK09_HUMA
175	MAPK3	mitogen-activated protein kinase 3	5595	MK03_HUMA
176	HMGB1	high mobility group box 1	3146	HMGB1_HUM
177	CCNA2	cyclin A2	890	CCNA2_HUM
178	HMGB2	high mobility group box 2	3148	HMGB2_HUM
179	MAP3K5	mitogen-activated protein kinase kinase	4217	M3K5_HUMA
180	TAF1	TAF1 RNA polymerase II, TATA box	6872	TAF1_HUMA
181	LMNB1	lamin B1	4001	LMNB1_HUM
182	SDHC	succinate dehydrogenase complex,	6391	C560_HUMAN
183	FOXO4	forkhead box O4	4303	FOXO4_HUM
184	HESX1	HESX homeobox 1	8820	HESX1_HUM
185	PIK3R1	phosphoinositide-3-kinase, regulatory	5295	P85A_HUMAN
186	BSCL2	Berardinelli-Seip congenital	26580	BSCL2_HUMA
187	AGPAT2	1-acylglycerol-3-phosphate O-	10555	PLCB_HUMA
188	BMI1	BMI1 proto-oncogene, polycomb ring	648	BMI1_HUMA
189	EEF1A1	eukaryotic translation elongation factor 1	1915	EF1A1_HUMA
190	TFAP2A	transcription factor AP-2 alpha	7020	AP2A_HUMA
191	BDNF	brain-derived neurotrophic factor	627	BDNF_HUMA
192	CREB1	cAMP responsive element binding	1385	CREB1_HUMA
193	ATF2	activating transcription factor 2	1386	ATF2_HUMA
194	TBP	TATA box binding protein	6908	TBP_HUMAN
195	APEX1	APEX nuclease (multifunctional DNA	328	APEX1_HUM
196	HBP1	HMG-box transcription factor 1	26959	HBP1_HUMA
197	BUB1B	BUB1 mitotic checkpoint	701	BUB1B_HUM
198	PTGS2	prostaglandin-endoperoxide synthase 2	5743	PGH2_HUMA
199	HSPA8	heat shock 70kDa protein 8	3312	HSP7C_HUMA

200	SIN3A	SIN3 transcription regulator family	25942	SIN3A_HUMA
201	CDK1	cyclin-dependent kinase 1	983	CDK1_HUMA
202	TFDP1	transcription factor Dp-1	7027	TFDP1_HUMA
203	DDIT3	DNA-damage-inducible transcript 3	1649	DDIT3_HUMA
204	POLA1	polymerase (DNA directed), alpha 1,	5422	DPOLA_HUM
205	MAPT	microtubule-associated protein tau	4137	TAU_HUMAN
206	CTGF	connective tissue growth factor	1490	CTGF_HUMA
207	HDAC2	histone deacetylase 2	3066	HDAC2_HUM
208	MAX	MYC associated factor X	4149	MAX_HUMA
209	MXD1	MAX dimerization protein 1	4084	MAD1_HUMA
210	MDM2	MDM2 proto-oncogene, E3 ubiquitin	4193	MDM2_HUMA
211	SUMO1	small ubiquitin-like modifier 1	7341	SUMO1_HUM
212	H2AFX	H2A histone family, member X	3014	H2AX_HUMA
213	HOXB7	homeobox B7	3217	HXB7_HUMA
214	HOXC4	homeobox C4	3221	HXC4_HUMA
215	JAK2	Janus kinase 2	3717	JAK2_HUMA
216	ESR1	estrogen receptor 1	2099	ESR1_HUMAN
217	LEP	leptin	3952	LEP_HUMAN
218	LEPR	leptin receptor	3953	LEPR_HUMA
219	NFKBIA	nuclear factor of kappa light polypeptide	4792	IKBA_HUMA
220	CLU	clusterin	1191	CLUS_HUMA
221	MTOR	mechanistic target of rapamycin	2475	MTOR_HUMA
222	GHRHR	growth hormone releasing hormone	2692	GHRHR_HUM
223	CTNNB1	catenin (cadherin-associated protein), beta	1499	CTNB1_HUM
224	PSEN1	presenilin 1	5663	PSN1_HUMA
225	DLL3	delta-like 3 (Drosophila)	10683	DLL3_HUMA
226	CDKN2A	cyclin-dependent kinase inhibitor 2A	1029	CDN2A_HUM
227	PPP1CA	protein phosphatase 1, catalytic subunit,	5499	PP1A_HUMA
228	DBN1	drebrin 1	1627	DREB_HUMA
229	NOG	noggin	9241	NOGG_HUMA
230	ELN	elastin	2006	ELN_HUMAN
231	ATR	ATR serine/threonine kinase	545	ATR_HUMAN
232	UCP3	uncoupling protein 3 (mitochondrial,	7352	UCP3_HUMA
233	ZMPSTE2	zinc metallopeptidase STE24	10269	FACE1_HUMA
234	TP63	tumor protein p63	8626	P63_HUMAN
235	UCP2	uncoupling protein 2 (mitochondrial,	7351	UCP2_HUMA
236	POLB	polymerase (DNA directed), beta	5423	DPOLB_HUM
237	GCLC	glutamate-cysteine ligase, catalytic	2729	GSH1_HUMA
238	GCLM	glutamate-cysteine ligase, modifier	2730	GSH0_HUMA
239	SIRT6	sirtuin 6	51548	SIRT6_HUMA

240	BUB3	BUB3 mitotic checkpoint protein	9184	BUB3_HUMA
241	RAE1	ribonucleic acid export 1	8480	RAE1L_HUMA
242	PMCH	pro-melanin-concentrating hormone	5367	MCH_HUMAN
243	MLH1	mutL homolog 1	4292	MLH1_HUMA
244	CSNK1E	casein kinase 1, epsilon	1454	KC1E_HUMA
245	STUB1	STIP1 homology and U-box containing	10273	CHIP_HUMAN
246	PPM1D	protein phosphatase, Mg2+/Mn2+	8493	PPM1D_HUM
247	CHEK2	checkpoint kinase 2	11200	CHK2_HUMA
248	PCK1	phosphoenolpyruvate carboxykinase 1	5105	PCKGC_HUM
249	ARHGAP	Rho GTPase activating protein 1	392	RHG01_HUM
250	CDC42	cell division cycle 42	998	CDC42_HUMA
251	ARNTL	aryl hydrocarbon receptor nuclear	406	BMAL1_HUM
252	CLOCK	clock circadian regulator	9575	CLOCK_HUM
253	HIC1	hypermethylated in cancer 1	3090	HIC1_HUMAN
254	PAPPA	pregnancy-associated plasma protein A,	5069	PAPP1_HUMA
255	ADCY5	adenylate cyclase 5	111	ADCY5_HUM
256	PPARGC	peroxisome proliferator-activated receptor	10891	PRGC1_HUM
257	GPX4	glutathione peroxidase 4	2879	GPX4_HUMA
258	UCP1	uncoupling protein 1 (mitochondrial,	7350	UCP1_HUMA
259	FGF23	fibroblast growth factor 23	8074	FGF23_HUMA
260	EFEMP1	EGF containing fibulin-like extracellular	2202	FBLN3_HUMA
261	ERCC4	excision repair cross-complementation	2072	XPF_HUMAN
262	CETP	cholesteryl ester transfer protein, plasma	1071	CETP_HUMA
263	PPARG	peroxisome proliferator-activated receptor	5468	PPARG_HUM
264	AGTR1	angiotensin II receptor, type 1	185	AGTR1_HUM
265	CISD2	CDGSH iron sulfur domain 2	493856	CISD2_HUMA
266	EEF1E1	eukaryotic translation elongation factor 1	9521	MCA3_HUMA
267	EPS8	epidermal growth factor receptor pathway	2059	ES8L2_HUMA
268	KCNA3	potassium channel, voltage gated shaker	3738	KCNA3_HUM
269	SIRT7	sirtuin 7	51547	SIR7_HUMAN
270	SLC13A1	solute carrier family 13 (sodium/sulfate	6561	A4D0X1_HUM
271	SOCS2	suppressor of cytokine signaling 2	8835	SOCS2_HUMA
273	TPP2	tripeptidyl peptidase II	7174	TPP2_HUMAN
274	TP53BP1	tumor protein p53 binding protein 1	7158	TP53B_HUMA
275	SIRT3	sirtuin 3	23410	SIR3_HUMAN
276	NCOR2	nuclear receptor corepressor 2	9612	NCOR2_HUM
277	SUN1	Sad1 and UNC84 domain containing 1	23353	SUN1_HUMA
278	BAK1	BCL2-antagonist/killer 1	578	BAK_HUMAN
279	IGFBP2	insulin-like growth factor binding protein	3485	IBP2_HUMAN
280	PYCR1	pyrroline-5-carboxylate reductase 1	5831	P5CR1_HUMA

281	TP73	tumor protein p73	7161	P73_HUMAN
282	CNR1	cannabinoid receptor 1 (brain)	1268	CNR1_HUMA
283	NFE2L2	nuclear factor, erythroid 2-like 2	4780	NF2L2_HUMA
284	CDKN1A	cyclin-dependent kinase inhibitor 1A	1026	CDN1A_HUM
285	PDGFRA	platelet-derived growth factor receptor,	5156	PGFRA_HUM
286	PIK3CA	phosphatidylinositol-4,5-bisphosphate 3-	5290	PK3CA_HUM
287	C1QA	complement component 1, q	712	C1QA_HUMA
288	CDKN2B	cyclin-dependent kinase inhibitor 2B	1030	CDN2B_HUM
289	EIF5A2	eukaryotic translation initiation factor	56648	IF5A2_HUMA
290	MIF	macrophage migration inhibitory factor	4282	MIF_HUMAN
291	DGAT1	diacylglycerol O-acyltransferase 1	8694	DGAT1_HUM
292	MT1E	metallothionein 1E	4493	MT1E_HUMA
293	FGF21	fibroblast growth factor 21	26291	FGF21_HUMA
294	HTRA2	HtrA serine peptidase 2	27429	HTRA2_HUM
295	GSK3A	glycogen synthase kinase 3 alpha	2931	GSK3A_HUM
296	NUDT1	nudix (nucleoside diphosphate linked	4521	8ODP_HUMA
297	IKBKB	inhibitor of kappa light polypeptide gene	3551	IKKB_HUMA
298	SQSTM1	sequestosome 1	8878	SQSTM_HUM
299	CDK7	cyclin-dependent kinase 7	1022	CDK7_HUMA
300	GRN	granulin	2896	GRN_HUMAN
301	SERPINE	serpin peptidase inhibitor, clade E (nexin,	5054	PAI1_HUMAN
302	SPRTN	SprT-like N-terminal domain	83932	SPRTN_HUM
303	RICTOR	RPTOR independent companion of	253260	RICTR_HUMA
304	CTF1	cardiotrophin 1	1489	CTF1_HUMAN
305	TRAP1	TNF receptor-associated protein 1	10131	TRAP1_HUMA
306	TRPV1	transient receptor potential cation channel	7442	TRPV1_HUMA
307	NFE2L1	nuclear factor, erythroid 2-like 1	4779	NF2L1_HUMA
308	IFNB1	Interferon beta	3456	IFNB_HUMAN
309	GDF11	growth differentiation factor 11	10220	A0A024RB20_
